# Phosphatase PPP1CC Regulates the First Lineage Segregation by GAS5 in Mouse Preimplantation Embryos

**DOI:** 10.1111/cpr.70155

**Published:** 2025-12-16

**Authors:** Jianwu Wang, Yan Zhang, Laijin Wu, Hongshuang Xie, Guang Yang, Yiwei Zhang, Cheng Huang, Shanyi Chou, Xuehan Li, Zhonghua Liu, Jiaqiang Wang

**Affiliations:** ^1^ Key Laboratory of Animal Cellular and Genetics Engineering of Heilongjiang Province, College of Life Science Northeast Agricultural University Harbin P. R. China

**Keywords:** hippo signalling pathway, mouse preimplantation embryo, phosphatase PPP1CC, reversible phosphorylation, subcortical localisation, the first lineage segregation

## Abstract

The transcriptional effector of the Hippo signalling pathway, YAP, regulates the first lineage specification in mouse preimplantation embryos. However, how YAP undergoes dephosphorylation specifically in the trophectoderm (TE) but not in the inner cell mass (ICM) remains unresolved. Here, we discovered that the serine/threonine phosphatase PPP1CC exhibits uniform distribution prior to blastocyst formation but becomes specifically localised to the TE during the blastocyst stage. Through mediating YAP dephosphorylation in the outer cells of mouse morula, PPP1CC facilitates YAP nuclear translocation, thereby ultimately driving TE lineage specification. Importantly, the spatially restricted localisation of PPP1CC in TE is achieved via its interaction with the long non‐coding RNA GAS5, which localises to the subcortical region throughout early mouse embryonic development. Knockdown of GAS5 phenocopies PPP1CC deficiency, causing developmental arrest at the morula stage accompanied by impaired YAP dephosphorylation in outer cells. Moreover, overexpression of GAS5 in one blastomere of the 2‐cell stage biases its descendants predominantly towards the TE fate. In summary, our study identifies the GAS5‐PPP1CC‐YAP axis as a central regulator of first lineage specification during mouse preimplantation development, highlighting its critical role in reversible phosphorylation during early embryogenesis.

## Graphical abstract

Subcortical GAS5 directs PPP1CC phosphatase spatiotemporal positioning during mouse morula‐blastocyst transition, controlling YAP dephosphorylation to drive first embryonic lineage specification.

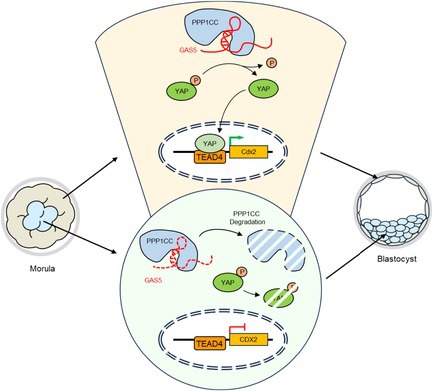

## 1 Introduction

The Hippo signalling pathway, initially discovered in *Drosophila melanogaster* and consisting of a cascade of kinases, DNA‐binding complexes, and transcriptional co‐activators, is highly conserved in both mice and humans [1, 2, 3, 4]. Core components of the mammalian Hippo pathway include Ste20‐like kinases 1/2 (MST1/2, homologues of Hpo), WW domain‐containing protein Salvador (SAV1, homologue of Sav), large tumour suppressors 1/2 (LATS1/2, homologues of Wts), their adaptor protein Mob as tumour suppressor (MOB1A/1B, homologues of Mats), the two Yki homologues Yes‐associated protein (YAP) and transcriptional co‐activator with PDZ‐binding motif (TAZ), and their downstream TEAD family transcription factors [5, 6, 7]. Upstream membrane protein receptors, upon sensing growth‐inhibitory signals from the extracellular environment, can activate the Hippo pathway [8]. This activation leads to the phosphorylation of Mst1/Mst2. The phosphorylated MST1/MST2 then directly phosphorylates the hydrophobic motifs of LATS1/2 (Lats1 T1079 and Lats2 T1041), which subsequently phosphorylates YAP [9]. Functioning in parallel with MST1/2, mitogen‐activated protein kinase kinase kinase kinases (MAP4Ks) family can also directly phosphorylate and activate the LATS1/2 kinases [10]. The phosphorylated YAP (p‐YAP) is anchored in the cytoplasm by 14‐3‐3 proteins and subsequently degraded by the proteasome [11].

The Hippo pathway is one of the major pathways regulating cell fate decisions in mammals and plays a central role during the first lineage segregation in mouse preimplantation embryos [12]. In the outer cells of the morula, the Hippo signalling pathway remains inactive. Consequently, unphosphorylated YAP accumulates in the nucleus, forms a complex with TEAD4, and induces the expression of CDX2 and other TE‐specific genes, ultimately promoting TE development [13, 14, 15]. Conversely, in the inner cells, Hippo signalling is activated, leading to p‐YAP being retained in the cytoplasm and degraded, resulting in the development of the inner cell mass [16]. However, previous studies have primarily focused on how different kinases regulate YAP localisation through phosphorylation at specific sites; the role of phosphatases in mediating YAP localisation remains poorly understood.

In this study, we found that the phosphatase PPP1CC is specifically localised in the TE of mouse blastocysts. Depletion of PPP1CC led to significant accumulation of p‐YAP in the outer cells of the morula, impairing their successful transition to the blastocyst stage. Mechanistically, subcortically localised GAS5 binds to the protein phosphatase PPP1CC, forming an RNA‐protein complex. This complex mediates the nuclear localisation of YAP in TE, a key downstream target gene of the Hippo signalling pathway, thereby regulating the fate of mouse preimplantation embryos. We also identified the critical binding sites between GAS5 and PPP1CC, and elucidated the essential role of PPP1CC in the Hippo signalling pathway. In summary, our findings demonstrate that the GAS5‐PPP1CC‐YAP axis is crucial for first lineage specification in mouse preimplantation embryos.

## 2 Materials and Methods

### 2.1 Ethics Statement

All the mouse procedures are carried out in compliance with the guidelines of the Animal Care and Use Committee of the Northeast Agricultural University.

### 2.2 Embryo Collection

Embryos were collected from 7‐week‐old ICR superovulated female mice crossed with ICR males, at the following times post‐human chorionic gonadotropin (phCG) injection: one‐cell stage (phCG 25 h), two‐cell stage (phCG 48 h), four‐cell stage (phCG 62 h), eight‐cell stage (phCG 74 h), morula stage (phCG 90 h), blastocyst stage (phCG 114 h).

### 2.3 Primer and Probe Design

All primers and probes were designed using Primer Premier 5 and synthesised from Comate Bioscience. Primers are shown in Table 1.

**TABLE 1:** Primers and probes.

Used for	Name	Sequence
qPCR	HPRT‐F	CCAGGTTATGACCTAGATTTG
	HPRT‐R	CAGAGGGCCACAATGTGATGG
	GAS5‐F1	AGCGTCGGGAATGGCAGTGT
	GAS5‐R1	CGTAAGCCTTCATCCTCCTTTGC
	GAS5‐F2	AATGGCAGTGTGGACCTCTGTGA
	GAS5‐R2	GAGTCCTCGTAAGCCTTCATCCTC
	GAS5‐F3	GCGCAATGTGCTAGAATAGAAGACC
	GAS5‐R3	GCCTTCACTTGAGGTGACCCAT
	CYR61‐F	AGATGCGGTTCCGATGCGAAGATG
	CYR61‐R	GTTGGGATGCGGGCAGTTGTAGTTA
	CTGF‐F1	ACCTGTGCCTGCCATTACAACTGTC
	CTGF‐R1	ACGCCATGTCTCCGTACATCTTCCT
	PPP1CC‐F	TGCGCCAGCATCAATAGGATCT
	PPP1CC‐R	CTTCTCGTCCACGATGGCTGC
	GAPDH‐F	GCAAATTCAACGGCACAGTCA
	GAPDH‐R	GCCTCACCCCATTTGATGTTAGT
	U6‐F	GCTTCGGCAGCACATATACTAAAAT
	U6‐R	CGCTTCACGAATTTGCGTGTCAT
FISH probes	GAS5‐FISH‐F1	GAGCTCGACAGCCCGGAGCCAA
	GAS5‐FISH‐R1	CAGCTCCTGCGGCGCTCAGTC
	GAS5‐FISH‐F2	CTCTGTGATGGGACATCTT
	GAS5‐FISH‐R2	GCGCTCGCTCTGTTATCCAGC
	GAS5‐FISH‐F3	AGGTATTAATGGGTCACCTCA
	GAS5‐FISH‐R3	TCCACTTGTCACAGGAGCC
siRNA	PPP1CC‐siRNA1	GGGACUGCUUUCCCUCAUU
	PPP1CC‐siRNA2	GGGCCCUCCUCCAUUUGAU
	Control‐siRNA	CAAGCUGACCCUGAAGUUCAU
LNA	lncGAS5‐LNA1	G‐C‐T‐g‐g‐a‐t‐a‐g‐a‐c‐a‐G‐T‐T‐T
	lncGAS5‐LNA2	G‐A‐C‐a‐g‐t‐t‐t‐g‐a‐a‐a‐G‐G‐T‐A
	Control‐LNA	A‐G‐A‐a‐c‐g‐g‐c‐a‐t‐c‐a‐A‐G‐G‐T

*Note*: In oligonucleotide sequences, uppercase letters denote LNA‐modified nucleotides, lowercase letters represent standard deoxynucleotides, and hyphens, indicate phosphorothioate backbone linkages (PTO).

### 2.4 Antibodies

The following antibodies were used for Western blot, Immunofluorescence and co‐IP assay: anti‐pYAP (Affinity Biosciences, AF3328), anti‐HA Tag (Abcam, ab1424), anti‐Flag Tag (Bioss, bsm‐33346M), anti‐YAP (Proteintech, 13584‐1‐AP), anti‐PPP1CC (SANTA CRUZ, sc‐515943), anti‐β‐Actin (Abcam, ab197345), anti‐CDX2 (Biogenex, MU392A‐UC), anti‐SOX2 (Santa Cruz, sc‐365823). Secondary antibodies: Donkey Anti‐Mouse IgG H&L (Alexa Fluor 488) (abcam, #ab150105), Goat Anti‐Rabbit IgG H&L (Alexa Fluor 488) (abcam, #ab150077), Goat Anti‐Rabbit IgG H&L (Alexa Fluor 647) (abcam, #ab150079), Rabbit Anti‐Mouse IgG H&L (Alexa Fluor 647) (abcam, #ab150127), HRP‐conjugated goat anti‐Rabbit IgG polyclonal secondary antibody (Invitrogen, A27036), and HRP‐conjugated rabbit anti‐Mouse IgG polyclonal secondary antibody (Invitrogen, 616520).

### 2.5 Microinjection

For GAS5 knockdown and mRNA overexpression, RNA (siRNA, 20 μM; mRNA, 150 ng·μL^−1^) was loaded into microneedles using a FemtoJet microinjector (Eppendorf, Germany). Equal volumes were injected per embryo under the following conditions: injection pressure 50 hPa, compensation pressure 20 hPa, and injection duration 0.3 s. Each embryo received approximately 5–10 pL of solution. Post‐injection, embryos were transferred to an incubator for cultivation.

### 2.6 Dual‐Luciferase Reporter Assay

NIH/3 T3 cells were co‐transfected with the following constructs: a reporter plasmid encoding firefly luciferase, the pRL‐TK plasmid expressing *Renilla* luciferase as a transfection control, and additional expression vectors specified in the Results section. After 48 h of incubation (with cell confluence maintained below 95%), the culture medium was aspirated. Cells were then washed with PBS under gentle agitation to remove non‐adherent cells and residual medium. This PBS washing step was repeated twice. Fluorescence intensity was quantified using the Dual‐Luciferase Reporter Assay System (Promega, E1910).

### 2.7 RNA Pull‐Down Assay

Utilising the Pierce Magnetic RNA‐Protein Pull‐Down Kit (Thermo Fisher, #20164), streptavidin magnetic beads were pre‐washed twice before incubation with biotinylated target RNA and cell lysates. After 30‐min room‐temperature binding with intermittent pipette mixing, the complexes underwent magnetic separation followed by two consecutive washes with 20 mM Tris buffer A. 100 μL Protein‐RNA binding mixture—comprising 10 μL Binding Buffer, 30 μL 50% glycerol, 30 μL additional salts, 30 μL lysate and nuclease‐free water—was added to the RNA‐bound beads and incubated at 4°C for 60 min. Post‐incubation magnetic separation preserved the supernatant, after which beads were washed three times with Wash Buffer. Target complexes were eluted in 50 μL Elution Buffer via vortex mixing, incubated at 37°C for 30 min, and the final supernatant collected for analysis.

### 2.8 Western Blot Analysis

Protein samples obtained from RNA pull‐down assays or extracted from embryos using Pierce IP Lysis Buffer (Thermo Fisher, #87787) were mixed with sample buffer and stored at −80°C. For analysis, 20 μL aliquots were thawed on ice, combined with 5 μL SDS‐PAGE Loading Buffer, and denatured at 100°C for 5 min. Following separation gel preparation and addition of electrophoresis buffer, samples (10 μL/lane) were resolved at constant 110 V for 60 min. Post‐electrophoresis, gels were trimmed and rinsed with deionised water. Proteins were transferred to PVDF membranes using a semi‐dry transfer system at 350 mA for 60 min with six filter papers and PVDF membranes assembled in transfer cassettes. Membranes were blocked with 3% BSA for 1 h at room temperature, then incubated with primary antibodies at 4°C overnight. After three 5‐min TBST washes, membranes were treated with secondary antibodies for 1 h at room temperature, followed by another series of TBST washes. Target proteins were detected by chemiluminescence after 1‐min substrate incubation (ECL reagent) and imaged using a chemiluminescence detection system.

### 2.9 RNA Extraction, Reverse Transcription, and In Vitro Transcription

Total RNA was isolated using TRIzol reagent, followed by DNase treatment with the RNase‐Free DNase Set (QIAGEN, #79254) to eliminate genomic DNA contamination. Reverse transcription was subsequently performed using RevertAid Reverse Transcriptase (Thermo Fisher, #EP0451). For in vitro mRNA synthesis, the HiScribe T7 ARCA mRNA Kit (NEB, #E2060S) was employed: A 20 μL capped transcription reaction mixture was assembled and incubated at 37°C for 30 min, followed by template DNA degradation through DNase I treatment (2 μL, 37°C, 15 min). Polyadenylation was then conducted at 37°C for 45 min, with final RNA purification achieved via 25 μL lithium chloride precipitation.

### 2.10 Quantitative Real‐Time PCR Analysis

Total RNA was extracted from collected embryos and reverse‐transcribed into cDNA, which was stored at −20°C until analysis. Gene expression quantification was performed using TB Green Premix Ex Taq (Takara Bio, RR420A) on a real‐time PCR system under the following thermal profile: initial denaturation at 95°C for 30 s, 40 cycles of denaturation (95°C, 5 s) and annealing/extension (60°C, 34 s), followed by a dissociation stage (95°C for 15 s and 60°C for 60 s). All reactions were conducted in triplicate, with target gene expression normalised to reference genes using the 2^(−ΔΔCt) method for relative quantification.

### 2.11 Immunofluorescence Staining

Embryos at various developmental stages were washed three times in PBS, treated with acidic Tyrode's solution to remove the zona pellucida, and rewashed in PBS. Samples were fixed in 4% paraformaldehyde for 30 min at room temperature, permeabilised in 1% Triton X‐100 for 30 min, and blocked with 3% BSA for 1 h. Primary antibody incubation was performed overnight at 4°C, followed by five 5‐min washes in antibody wash buffer. Secondary antibodies were applied for 1 h, succeeded by another five 5‐min washes. Embryos were mounted in antifade medium containing 5 μL nuclear counterstain (Thermo Fisher, P36971) and stored at 4°C protected from light.

### 2.12 RNA‐Fish

Perform the assay using a Type G in situ hybridisation detection kit (Focofish, D‐0010). Embryos underwent fixation in 4% paraformaldehyde. Pre‐hybridisation involved sequential treatments: Solution A (37°C, 15 min), Solution B (37°C, 15 min), three PBS washes, Solution C (37°C, 20 min), three additional PBS washes, 3% H₂O₂ incubation (RT, 15–20 min), and final fixation in 4% PFA (RT, 10 min) followed by three PBS rinses. After complete air‐drying, Cy3‐UTP‐labelled probes were denatured (85°C, 4 min) in mRNA Hybridisation Buffer and equilibrated (37°C, 2 min). Hybridisation was performed with 50 μL probe mixture (37°C, 18 h). Post‐hybridisation washes included: 1× Washing Buffer (37°C, 10 min) and fresh buffer (RT, 5 min). Nuclei were counterstained with DAPI‐antifade solution before mounting and imaging.

### 2.13 RNA Immunoprecipitation (RIP) Assay

Perform the assay using a RNA Binding Protein Immunoprecipitation (RIP) Kit (Sangon Biotech, B605109‐0006). Embryos were washed in PBS and lysed in 405 μL Complete RIP Lysis Buffer (400 μL RIP Lysis Buffer +4 μL 100 × Protease Inhibitor +1 μL RNase Inhibitor). One‐quarter of the lysate was reserved as input control for RNA extraction. The remaining lysate was resuspended in 400 μL fresh Complete RIP Lysis Buffer, vortexed every 10 min during 30‐min ice incubation, and centrifuged (12,000 rpm, 10 min, 4°C). The supernatant was aliquoted into: 150 μL (IP), 150 μL (IgG control), and 100 μL (input; stored at −80°C).

Protein A/G Magnetic Beads (30 μL per sample) were washed thrice in 300 μL RIP Wash Buffer. Beads were incubated with 5 μg target antibody (IP) or species‐matched IgG (control) for 60 min at RT, followed by three washes. For immunoprecipitation, 900 μL RIP Buffer (1720 μL RIP Wash Buffer +70 μL 0.5 M EDTA +10 μL RNase Inhibitor) and 150 μL Lysis were added to antibody‐bound beads and incubated overnight at 4°C. After five stringent washes with RIP Wash Buffer, beads were split: Part A (100 μL): Eluted in 100 μL Elution Buffer, heat‐denatured (100°C, 10 min), mixed with 6× Loading Buffer for western blot. Part B (200 μL): Subjected to RNA purification for RT‐qPCR/sequencing. The raw sequencing data were processed using Trim Galore (v0.6.7) for quality control and adapter trimming to generate clean reads. The clean data were then aligned and quantified by Salmon (v0.11.3) for transcript‐level analysis. Diferentially expressed genes (DEGs) were identified using DESeg2 with the thresholds of adjusted *p*‐value (padj) < 0.01 and fold change (FC) > 2.

### 2.14 Co‐Immunoprecipitation (Co‐IP) Assay

293T cells were co‐transfected with MS2P‐HA and GAS5‐24 × MS2 vectors using Lipofectamine LTX & PLUS Reagent (Thermo Fisher, #15338100). For Co‐IP, the Pierce Crosslink Magnetic IP/Co‐IP Kit (Thermo Fisher, #88805) with HA antibodies crosslinked to Protein A/G Magnetic Beads was employed. Transfected cells were lysed in 100 μL IP Lysis Buffer, with 10 μL lysate reserved as input control. The remaining lysate underwent overnight incubation at 4°C with anti‐HA‐conjugated beads. After two 5‐min washes with IP Lysis Buffer, beads were resuspended in 500 μL ultrapure water. Target complexes were eluted in 100 μL Elution Buffer, mixed with 15 μL 6 × SDS loading buffer, and heat‐denatured at 100°C for 5 min. Co‐precipitated PPP1CC and HA‐tagged proteins were detected by western blot.

### 2.15 Image Analysis

For fluorescence intensity analysis of embryos, quantitative fluorescence measurements were performed using Leica LAS X and Fiji software [17]. For each embryo, the maximum projection was acquired using LAS X. For the analysis of the nucleo‐cytoplasmic signal intensity ratio, the nuclear region and a cytoplasmic region of equal size were cropped using the Fiji region of interest (ROI) function, and the mean signal intensities were extracted. To normalise the signals to the DAPI fluorescence level, the Fiji ROI function was used to extract the nuclear region stained for the specific proteins and the corresponding DAPI channel region. Normalisation was performed using the formula: target protein signal/DAPI signal.

### 2.16 Preparation of Mutant

Site‐directed mutagenesis was performed using the QuickMutation Gene Mutagenesis Kit according to the following procedure: A 50 μL PCR reaction mixture was prepared containing 0.4 μM primers, 200 ng of the target plasmid template, 5 μL of 10× BeyoFusion Buffer (with Mg^2+^), 0.25 mM dNTP mix, and 1 μL of BeyoFusion DNA Polymerase. Following amplification, 1 μL of DpnI restriction enzyme was directly added to the reaction mixture and incubated at 37°C for 5 min. The digested product was subsequently transformed into competent bacterial cells for overnight culture. All sequences underwent validation through Sanger sequencing by Comate Bioscience prior to their application.

### 2.17 Data Analysis

Statistical analyses [mean ± standard error of the mean (SEM)] for differential gene expression, differential fluorescence intensity, and differential abundance on gels were performed in Excel. Levels of significance were calculated with Student's *t*‐tests. Isoform abundance on SDS‐PAGE gels or agarose gels was measured in Fiji/ImageJ.

## 3 Results

### 3.1 PPP1CC Promotes YAP Transcriptional Activity

**FIGURE 1:**
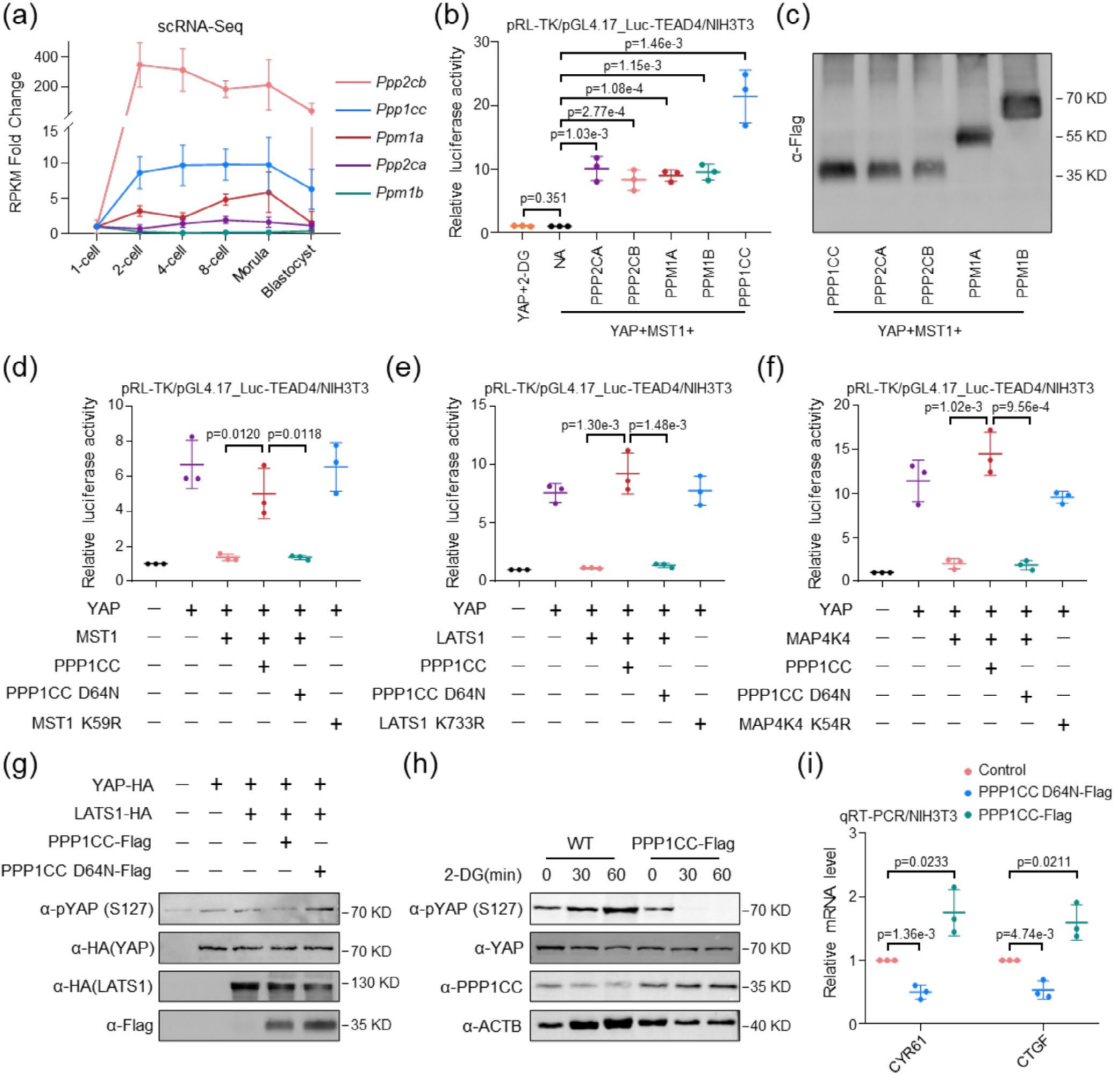
Phosphatase PPP1CC promotes YAP transcriptional activity. (a) Line plots showing the mRNA profiles of *Ppp2cb*, *Ppp1cc*, *Ppm1a*, *Ppp2ca* and *Ppm1b* in various developmental stages of mouse preimplantation embryos were examined by RNA‐seq (Single‐cell RNA‐seq data source GSE45719). (b) YAP‐driven transcriptional activity from the TEAD4‐responsive promoter was strongly suppressed by co‐expression of MST1, an upstream kinase in the Hippo pathway. Screening five known phosphatases in NIH/3T3 cells identified PPP1CC as the most effective at restoring YAP‐TEAD4 transcriptional activation inhibited by MST1. *n* = 3 biologically independent experiments. Two‐tailed Student's *t*‐test was used for the statistical analysis. The data are presented as the mean ± SEM. (c) Western blot detection of Flag‐tagged phosphatases in NIH/3 T3 cells. Five known phosphatases were screened for expression levels via immunoblotting against the Flag‐tag. *n* = 3 biologically independent experiments. α‐, anti‐. (d–f) Co‐expression of Hippo pathway upstream factors MST1 (d), LATS1 (e), and MAP4K4 (f) suppressed YAP transcriptional activity. Co‐transfection of wild‐type PPP1CC significantly restored YAP‐driven TEAD4 activity, while the phosphatase‐dead PPP1CC (D64N) mutant failed to restore transcriptional activity. *n* = 3 biologically independent experiments. Two‐tailed Student's *t*‐test was used for the statistical analysis. The data are presented as the mean ± SEM. (g) LATS1‐induced phosphorylation of YAP was abolished by co‐transfection of phosphatase PPP1CC, but not by the phosphatase‐dead PPP1CC mutant. *n* = 3 biologically independent experiments. α‐, anti‐. (h) Under 2‐DG‐induced energy stress, YAP phosphorylation levels increased with treatment duration. However, overexpression of PPP1CC significantly attenuated p‐YAP levels. *n* = 3 biologically independent experiments. α‐, anti‐. (i) PPP1CC induction significantly increased mRNA levels of YAP target genes CYR61 and CTGF. Expression of CTGF and CYR61 was detected by quantitative real‐time PCR (qRT‐PCR). *n* = 3 biologically independent experiments. Two‐tailed Student's *t*‐test was used for the statistical analysis. The data are presented as the mean ± SEM.

Previously published data indicated that five phosphatases, PPM1A/PP2Cα (Protein Phosphatase, Mg^2+^/Mn^2+^ dependent 1A), PPP2CA/PP2Aα (Protein Phosphatase 2 Catalytic subunit alpha), PPP1CC/PP1γ (Protein Phosphatase 1 Catalytic subunit gamma), PPP2CB/PP2Aβ (Protein Phosphatase 2 Catalytic subunit beta), and PPM1B/PP2Cβ (Protein Phosphatase, Mg^2+^/Mn^2+^ dependent 1B) could reverse YAP activity following MST1 stimulation in 293T cells [18]. To investigate whether these phosphatases regulate the Hippo‐YAP signalling pathway in mouse embryos, we first analysed the expression changes of these five phosphatases in mouse preimplantation embryos using public single‐cell transcriptome data [19]. *Ppp2cb* exhibits a dramatic increase in expression at the 2‐cell stage, then gradually decreased while remaining relatively high until the blastocyst (Figure 1a). *Ppp1cc* expression also changed significantly, progressively increasing from the zygote to the 8‐cell stage before slightly declining. In contrast, the other three phosphatases showed relatively low expression levels during early embryonic development (Figure 1a). Besides, we analysed their effect on YAP‐TEAD4 transcriptional activity under conditions of MST1‐induced Hippo signalling activation using mouse embryonic fibroblasts (MEFs). Dual‐luciferase reporter assays revealed that all five phosphatases enhanced YAP‐TEAD4 transcriptional activity, which was suppressed by MST1 (Figure 1b,c and Figure S1a,b). Notably, the phosphatase PPP1CC exhibited a significantly stronger (about two‐fold higher) ability to reverse the inhibitory effect of MST1 compared to the other four phosphatases (Figure 1b).

Based on the striking effect of PPP1CC on promoting YAP‐TEAD4 activity and its dynamic expression pattern during early embryonic development, we selected PPP1CC for further investigation. We constructed mutant vectors of PPP1CC and its upstream kinase factors to delineate its functional role in the Hippo pathway. Reporter assays demonstrated that PPP1CC could attenuated the inhibition of YAP driven by the core Hippo kinases MST1 and LATS1, leading to increased activity of the TEAD4‐responsive promoter (Figure 1d,e). PPP1CC was also able to reverse YAP inhibition driven by MAP4K4, another upstream kinase of LATS1 [10] (Figure 1f). The regulation of Hippo signalling by PPP1CC critically depends on its phosphatase activity, as the phosphatase‐dead PPP1CC mutant (D64N) failed to enhance YAP‐driven TEAD4 activity (Figure 1d–f). Additionally, we examined the phosphorylation levels of YAP and LATS1 following PPP1CC overexpression. The results showed a decrease in p‐YAP levels, while p‐LATS1 remained unchanged and was detected at low expression levels, indicating that PPP1CC regulates YAP phosphorylation independently of LATS1 (Figure S1c).

YAP phosphorylation represents a critical regulatory modification. p‐YAP binds to cytoplasmic proteins and is retained in the cytosol, where it subsequently undergoes ubiquitination and degradation. This process suppresses YAP‐mediated pro‐growth and anti‐apoptotic functions [20]. Accordingly, we detected p‐YAP expression and demonstrated that PPP1CC significantly attenuates p‐YAP activity (Figure 1g and Figure S1d). Furthermore, we validated the role of PPP1CC under physiological stimulation using 2‐deoxyglucose (2‐DG, a glucose analog and metabolic inhibitor) [21, 22, 23]. As anticipated, PPP1CC alleviated 2‐DG‐induced energy stress leading to YAP phosphorylation (Figure 1h and Figure S1e).

Consistent with these observations, we detected significantly elevated mRNA expression levels of *Cyr61* and *Ctgf*, two well‐established target genes of the YAP‐TEAD4 transcriptional complex, upon PPP1CC overexpression [24, 25, 26]. Conversely, inactivation of PPP1CC phosphatase activity significantly suppressed the transcription of *Cyr61* and *Ctgf* (Figure 1i). These findings indicate that PPP1CC can eliminate critical phosphorylation modifications on YAP, thereby influencing Hippo signalling pathway activity.

### 3.2 PPP1CC Depletion Leads to Developmental Arrest at Morula Stage

**FIGURE 2:**
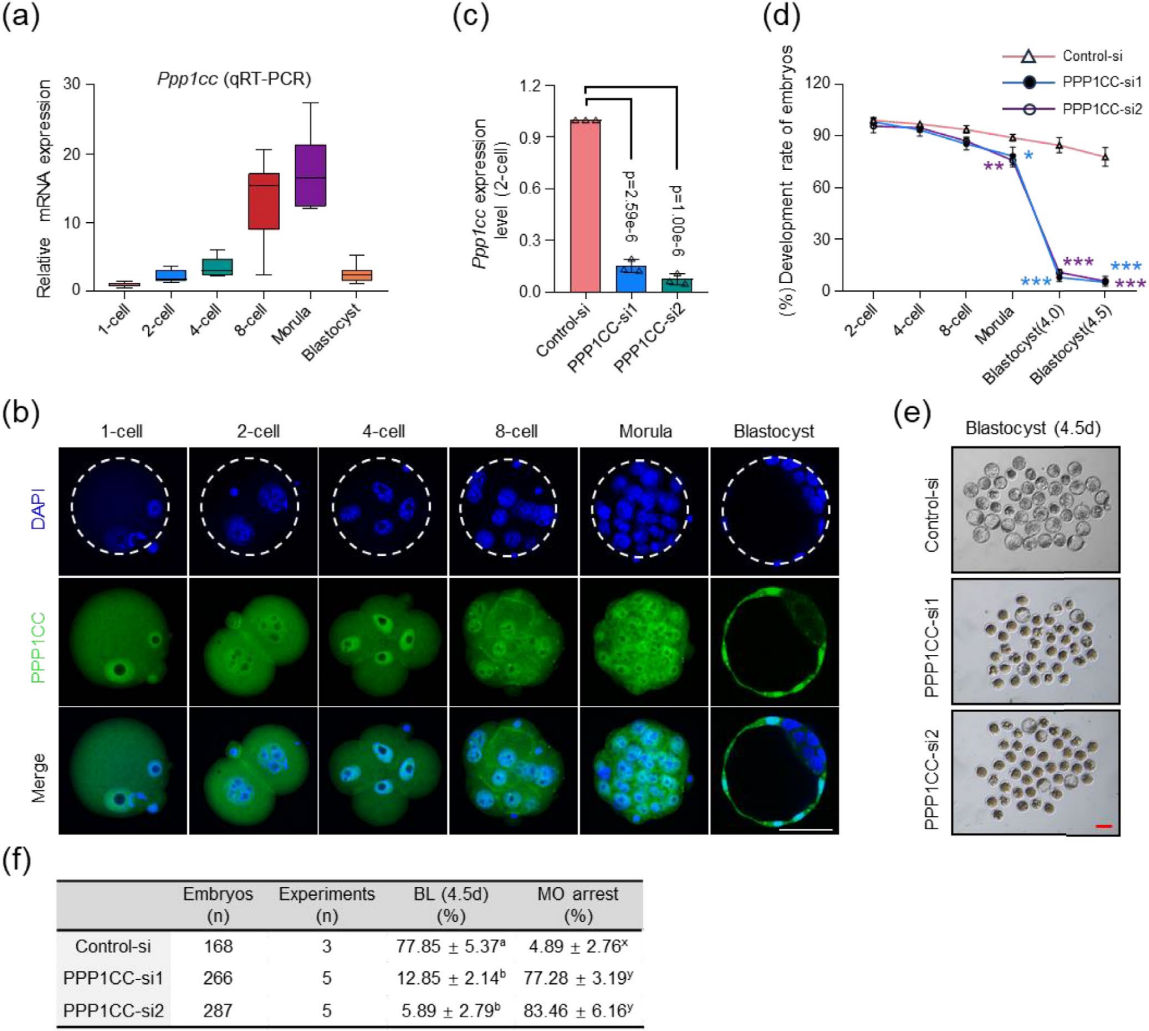
Depletion of PPP1CC causes embryonic arrest at the morula stage in mice. (a) qRT‐PCR analysis of relative *Ppp1cc* mRNA expression in embryos at different developmental stages. *n* = 3 biologically independent experiments, using Hprt as the reference gene. Box plots show medians (center lines) and interquartile ranges (box edges). (b) Immunofluorescence staining of PPP1CC in embryos at different developmental stages. Sample sizes 1‐cell (*n* = 11), 2‐cell (*n* = 12), 4‐cell (*n* = 11), 8‐cell (*n* = 13), morula (*n* = 18), blastocyst (*n* = 15). ‘*n*’ denotes the number of embryos per group. Scale bar: 50 μm. (c) Effective knockdown of Ppp1cc mRNA mediated by RNAi. siRNA was injected at 25 h post‐hCG (phCG), and late 2‐cell embryos were collected at 48 h phCG for qRT‐PCR analysis. Approximately 100 embryos per stage were used across *n* = 3 biologically independent experiments. Two‐tailed Student's *t*‐test was used for the statistical analysis. The data are presented as the mean ± SEM. (d) Statistical analysis of the effect of PPP1CC siRNA injection on embryonic development rates. *n* = 3 biologically independent experiments. Two‐tailed Student's *t*‐test was used for the statistical analysis. The data are presented as the mean ± SEM. PPP1CC‐si1 (blue line) versus Control‐si (pink line) 2‐cell (*p* = 0.618), 4‐cell (*p* = 0.157), 8‐cell (*p* = 0.111), morula (*p* = 0.0142), blastocyst 4.0d (*p* = 1.06e‐7), blastocyst 4.5d (*p* = 2.69e‐7). PPP1CC‐si2 (purple line) vs. Control‐si (pink line) 2‐cell (*p* = 0.222), 4‐cell (*p* = 0.211), 8‐cell (*p* = 0.0900), morula (*p* = 3.86e‐3), blastocyst 4.0d (*p* = 2.92e‐7), blastocyst 4.5d (*p* = 2.34e‐7). *p < 0.05; **p < 0.01; ***p < 0.001. (e) Depletion of PPP1CC causes embryonic arrest at the morula stage. siRNA was injected at 25 h phCG, and late blastocysts were imaged at 114 h phCG (4.5d). Control‐injected embryos developed to the late blastocyst stage, whereas PPP1CC‐depleted embryos failed to progress beyond the morula stage. *n* = 3 biologically independent experiments. Scale bar 100 μm. (f) Embryonic development following PPP1CC siRNA injection. MO Morula; BL Blastocyst. siRNA concentration for both control and knockdown groups was 150 ng/μL. Different letters within the same column indicate statistically significant differences (*p* < 0.001)

Studies in male mice show that PPP1CC plays a crucial role in testis development and male gametogenesis [27]. PPP1CC‐deficient mice exhibit abnormalities in sperm motility, differentiation, and morphogenesis in the testes [28, 29]. However, whether PPP1CC regulates the Hippo signalling pathway via dephosphorylation in mouse preimplantation embryos remained to be investigated. qRT‐PCR analysis confirmed that the expression pattern of *Ppp1cc* in preimplantation embryos was consistent with the RNA sequencing results [19] (Figure 2a). Immunofluorescence (IF) analysis revealed that PPP1CC exhibits uniform distribution in embryos from the 1‐cell to morula stages (Figure 2b). However, during the blastocyst stage, it becomes predominantly localised to the outer cells (Figure 2b). This TE‐specific distribution in the blastocyst suggests a potential role in regulating lineage differentiation.

To probe the involvement of PPP1CC in early mouse embryo development, we injected interfering fragments targeting PPP1CC at the 1‐cell stage. Successful PPP1CC knockdown was verified through qRT‐PCR, Western blotting, and IF analyses (Figure 2c and Figure S2a–c). Results demonstrated that embryonic developmental progression remained unaffected prior to morula formation following PPP1CC knockdown (Figure 2d). But developmental arrest occurred predominantly at the morula stage, with blastocyst formation observed in merely a fraction of embryos (Figure 2e,f and Figure S2d). Additionally, PPP1CC overexpression did not significantly alter the early embryonic developmental rate (Figure S2e). These results demonstrate the critical importance of PPP1CC for the morula‐to‐blastocyst transition in mouse preimplantation embryos and provide further support for the involvement of PPP1CC in regulating the first lineage segregation.

### 3.3 Depletion of PPP1CC Impairs YAP Dephosphorylation in Morula

Given the promotive effect of PPP1CC on Hippo‐YAP activity observed in the above‐described cell‐based study, we hypothesised that the developmental arrest at the morula stage caused by PPP1CC knockdown might be due to impaired Hippo‐YAP signalling. To test this hypothesis, we performed IF staining on PPP1CC depleted embryos arrested at the morula stage (Figure S3a).

**FIGURE 3:**
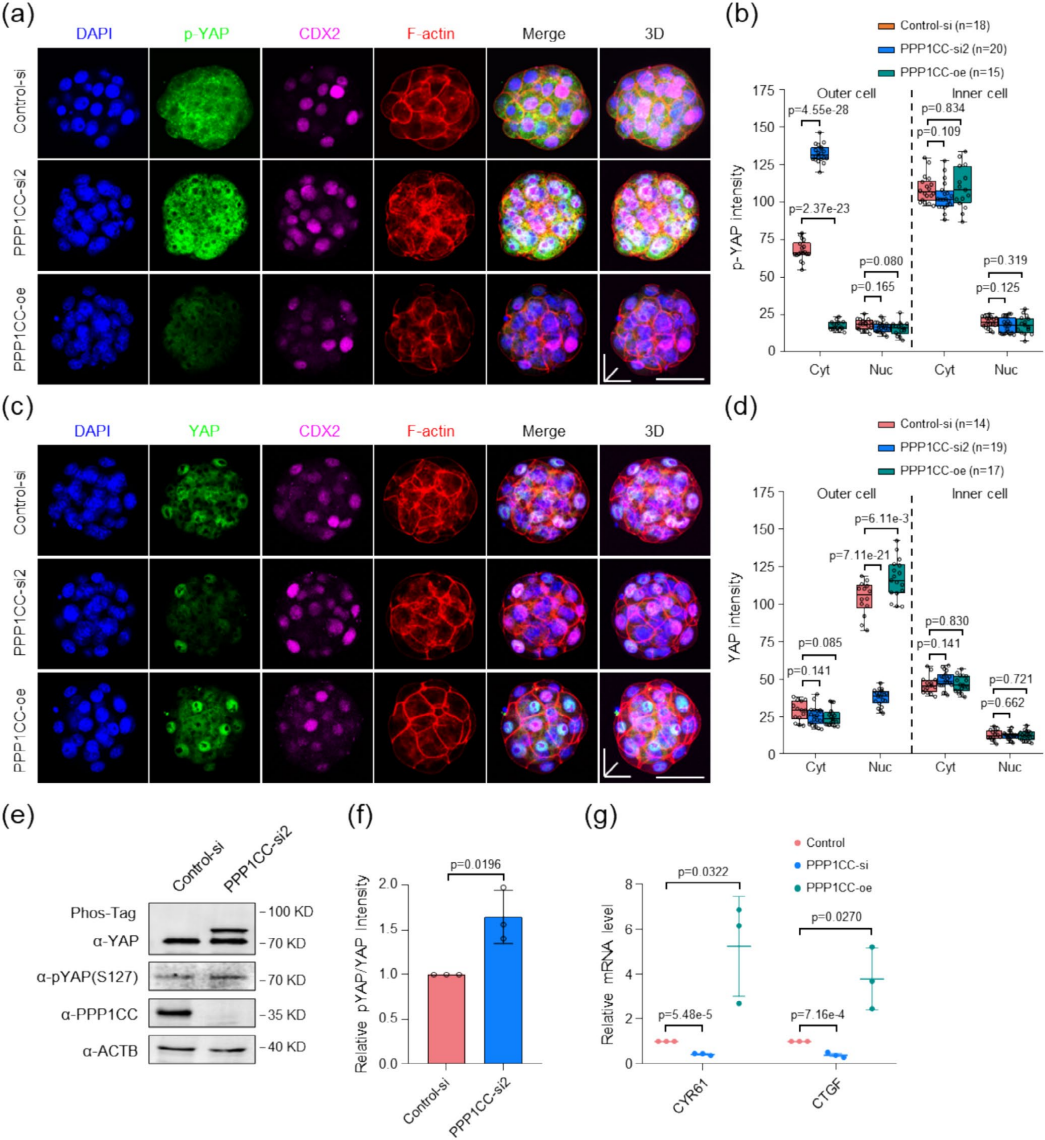
p‐YAP accumulates in the outer cells of morula arrested due to PPP1CC knockdown. (a) Representative immunofluorescence staining images of p‐YAP and CDX2 in control (Control‐si), PPP1CC knockdown (PPP1CC‐si2), and PPP1CC overexpressing (PPP1CC‐oe) morula. Embryos were collected at 90 h post‐hCG (phCG). Images are representative of three independent experiments. Scale bar: 50 μm. (b) Quantification of fluorescence intensity. Morula were divided into outer and inner cells. Fluorescence intensity of p‐YAP in the cytoplasm (Cyt) and nucleoplasm (Nuc) was quantified separately for outer and inner cells. *n =* 18, 20, and 15 embryos representing three independent biological replicate experiments. ‘*n*’ denotes the number of embryos per group. Two‐tailed Student's *t*‐test was used for the statistical analysis. The data are presented as the mean ± SEM. (c) Representative immunofluorescence staining images of YAP and CDX2 in control (Control‐si), PPP1CC knockdown (PPP1CC‐si2), and PPP1CC overexpressing (PPP1CC‐oe) morula. Embryos were collected at 90 h phCG. Images are representative of three independent experiments. Scale bar: 50 μm. (d) Quantification of fluorescence intensity. Morula were divided into outer and inner cells. Fluorescence intensity of YAP in the cytoplasm (Cyt) and nucleoplasm (Nuc) was quantified separately for outer and inner cells. *n =* 14, 19, and 17 embryos representing three independent biological replicate experiments. ‘*n*’ denotes the number of embryos per group. Two‐tailed Student's *t*‐test was used for the statistical analysis. The data are presented as the mean ± SEM. (e) Increased YAP phosphorylation levels in PPP1CC‐knockdown embryos, as revealed by mobility shift in Phos‐tag gel electrophoresis. siRNA or control was injected at 25 h phCG, and morulae were collected at 90 h phCG for immunoblot analysis. Approximately 300 embryos per group were used across three experimental replicates. (f) Protein intensities were normalised. Phosphorylation levels are represented as the ratio of phosphorylated protein to total protein. *n* = 3 biologically independent experiments. Two‐tailed Student's *t*‐test was used for the statistical analysis. The data are presented as the mean ± SEM. (g) PPP1CC promotes expression of YAP target genes *CTGF* and *CYR61*. Control‐si, PPP1CC‐si2, or PPP1CC‐oe constructs were injected at 25 h phCG. Morula were collected at 90 h phCG, and expression of *CTGF* and *CYR61* was detected by qRT‐PCR. Approximately 100 embryos per group were used across *n* = 3 biologically independent experiments. Two‐tailed Student's *t*‐test was used for the statistical analysis. The data are presented as the mean ± SEM.

Intriguingly, PPP1CC ablation led to a significant accumulation of p‐YAP in the cytoplasm of outer cells in the morula (Figure 3a,b). This phenotype could be rescued by overexpression of PPP1CC (Figure 3a,b). We also examined changes in total YAP expression. The results showed that nuclear‐YAP expression was significantly reduced in the outer cells following PPP1CC knockdown (Figure 3c,d). Similarly, this phenotype could be rescued by PPP1CC overexpression (Figure 3c,d). Moreover, PPP1CC overexpression also leads to an increase in the number of CDX2‐positive cells (Figure S3b). When development reaches the blastocyst stage, although the total cell number remains unchanged, the ICM/TE ratio is decreased (Figure S3c,d). These findings indicate that when PPP1CC function is impaired in morulae, YAP in outer cells fails to be dephosphorylated and translocate to the nucleus.

We collected morula embryos developmentally arrested due to PPP1CC knockdown. Phos‐tag gel electrophoresis revealed a significantly greater mobility shift of endogenous YAP upon PPP1CC depletion, indicating elevated levels of p‐YAP (Figure 3e). Furthermore, we observed an overall increase in p‐YAP levels following PPP1CC knockdown (Figure 3f). Interestingly, the level of p‐YAP in embryos upon PPP1CC knockdown was comparable to that observed in cells expressing the phosphatase‐dead PPP1CC mutant (D64N), suggesting that PPP1CC similarly regulates the Hippo‐YAP signalling pathway in mouse early embryos (Figure 3f and Figure S1d).

Moreover, we assessed the expression levels of downstream target genes of YAP. As predicted, *Cyr61* and *Ctgf* expression decreased upon PPP1CC knockdown but increased following PPP1CC overexpression (Figure 3g). These results demonstrate that the phosphatase PPP1CC mediates YAP dephosphorylation in mouse morula embryos.

### 3.4 PPP1CC Is Specifically Localised to the TE

**FIGURE 4:**
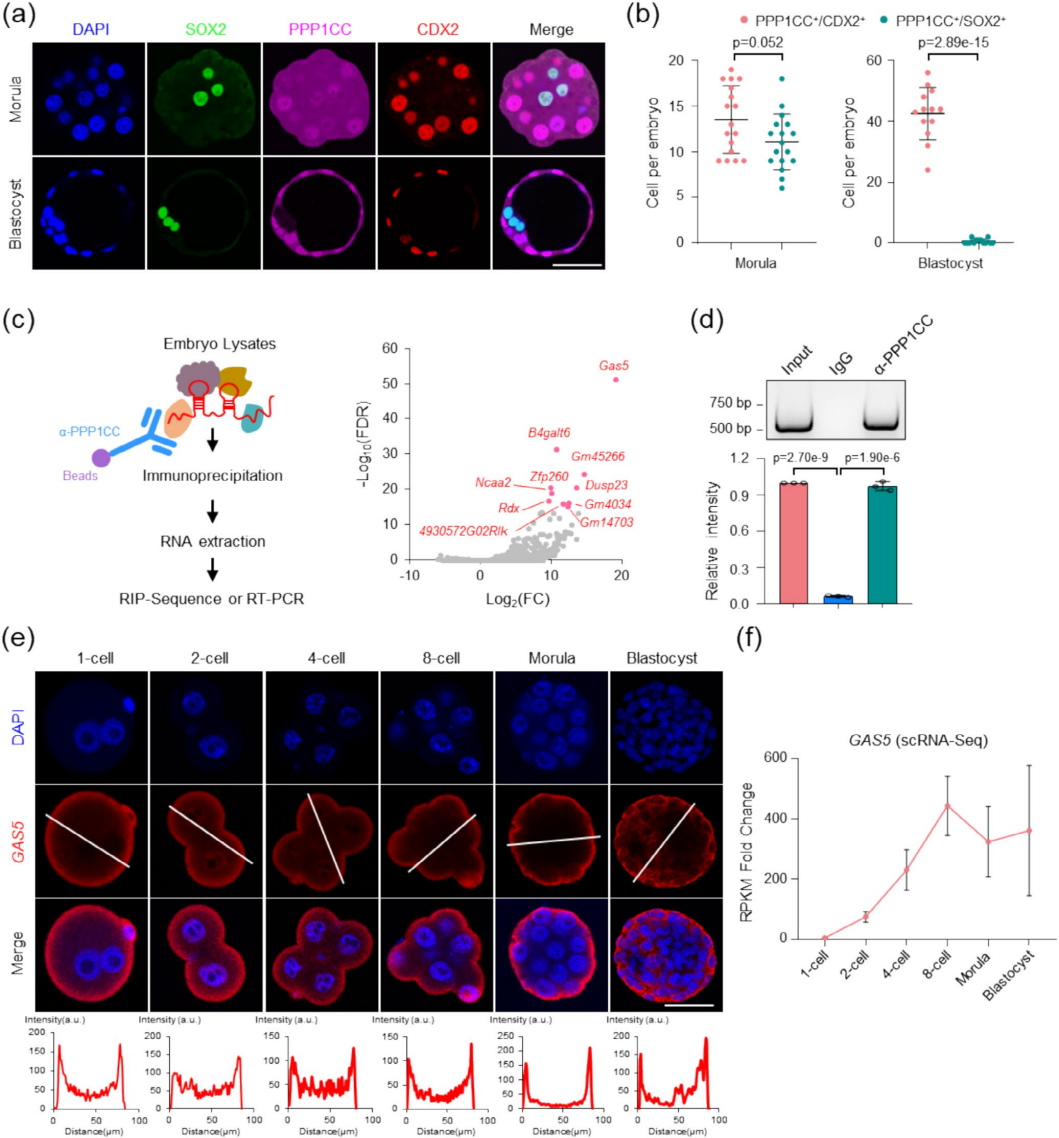
PPP1CC localisation to TE accomplished by subcortical GAS5. (a) Representative immunofluorescence staining images for CDX2, SOX2, and PPP1CC in morula and blastocysts. Images are representative of three independent experiments (Morula *n* = 17; Blastocyst *n* = 10). ‘*n*’ denotes the number of embryos per group. Scale bar 50 μm. (b) Quantification of PPP1CC‐positive cells in parallel with CDX2‐positive and SOX2‐positive cell counts at the morula and blastocyst stages. PPP1CC^+^ denotes PPP1CC‐positive cells, CDX2^+^ denotes CDX2‐positive cells, SOX2^+^ denotes SOX2‐positive cells. Two‐tailed Student's *t*‐test was used for the statistical analysis. The data are presented as the mean ± SEM. (c) RNAs bound to PPP1CC were identified by RIP‐seq. scatter plot shows differentially enriched RNAs in PPP1CC immunoprecipitates. Red dots indicate the top 10 enriched RNAs, with lncRNA GAS5 being the most significantly enriched (fold change > 2, FDR < 0.05). FC, Fold Change, FDR, False Discovery Rate. (d) PPP1CC RIP assays followed by qRT‐PCR detection of co‐precipitated GAS5 RNA (bottom panel). Agarose gel electrophoresis of PCR products (upper panel). *n* = 3 biologically independent experiments. Two‐tailed Student's *t*‐test was used for the statistical analysis. The data are presented as the mean ± SEM. (e) RNA‐FISH detection of GAS5 in 1‐cell to blastocyst‐stage embryos. GAS5 persists throughout mouse preimplantation development with predominant subcortical localisation and minimal nuclear signal. Sample sizes 1‐cell (*n* = 11), 2‐cell (*n* = 10), 4‐cell (*n* = 11), 8‐cell (*n* = 10), morula (*n* = 13), blastocyst (*n* = 12). ‘*n*’ denotes the number of embryos per group. Scale bar 50 μm. Three experimental replicates were performed. (f) Expression profiling of lncRNA GAS5 across developmental stages of mouse preimplantation embryos was performed using RNA‐seq (Single‐cell RNA‐seq data source GSE45719). Data are presented as mean values ± SEM. Statistical analysis was performed using two‐tailed unpaired *t*‐tests.

To further investigate the mechanisms underlying differential YAP regulation between the TE and ICM, we performed comprehensive analysis of PPP1CC spatial distribution in inner and outer cells during both morula and blastocyst stages. Co‐staining of PPP1CC with CDX2 and SOX2 confirmed its homogeneous distribution in morula embryos (Figure 4a,b). However, PPP1CC became exclusively localised to the TE at the blastocyst stage (Figure 4a,b).

### 3.5 PPP1CC Binds TE‐Localised GAS5

These results indicate that PPP1CC progressively localises to the TE during the morula‐to‐blastocyst transition. However, the precise molecular mechanisms underlying this process remain unresolved. It is well established that long non‐coding RNAs (lncRNAs) can regulate protein localisation and function, including *Neat1* [30] and *LincGET* [31]. We therefore wondered whether TE‐specific localisation of PPP1CC in the blastocyst is accomplished by lncRNA.

To investigate this, we performed RNA Immunoprecipitation Sequencing (RIP‐Seq) to screen for lncRNAs interacting with PPP1CC. GAS5 was raised by the RIP‐seq for its highest fold change (Figure 4c). qRT‐PCR and agarose gel electrophoresis further confirmed the binding between PPP1CC and GAS5 (Figure 4d). Moreover, research in cancer cells indicates that GAS5 stabilises p53 by recruiting p300 to bind p53, thereby repressing telomerase activity and inhibiting the proliferation of Laryngeal Squamous Cell Carcinoma (LSCC) cells [32]. We hypothesise that GAS5 may similarly regulate PPP1CC protein stability. To investigate the regulatory role of GAS5, we initially performed RNA fluorescence in situ hybridisation (RNA‐FISH) to determine the distribution of GAS5 in early embryos. Intriguingly, from the 1‐cell to the blastocyst stage, GAS5 consistently localised to the subcortical region (Figure 4e). Further subcellular localisation analysis confirmed cytoplasmic localisation of GAS5 (Figure S4a). This polarised distribution of GAS5 further underscores its potential regulatory role on TE‐specific localisation of PPP1CC in blastocyst. Comprehensive profiling of GAS5 expression across mouse preimplantation stages demonstrated spatiotemporal concordance with PPP1CC dynamics, as validated by RNA‐Seq and qRT‐PCR (Figure 4f and Figure S4b).

### 3.6 Key Sequence for GAS5‐PPP1CC Binding

**FIGURE 5:**
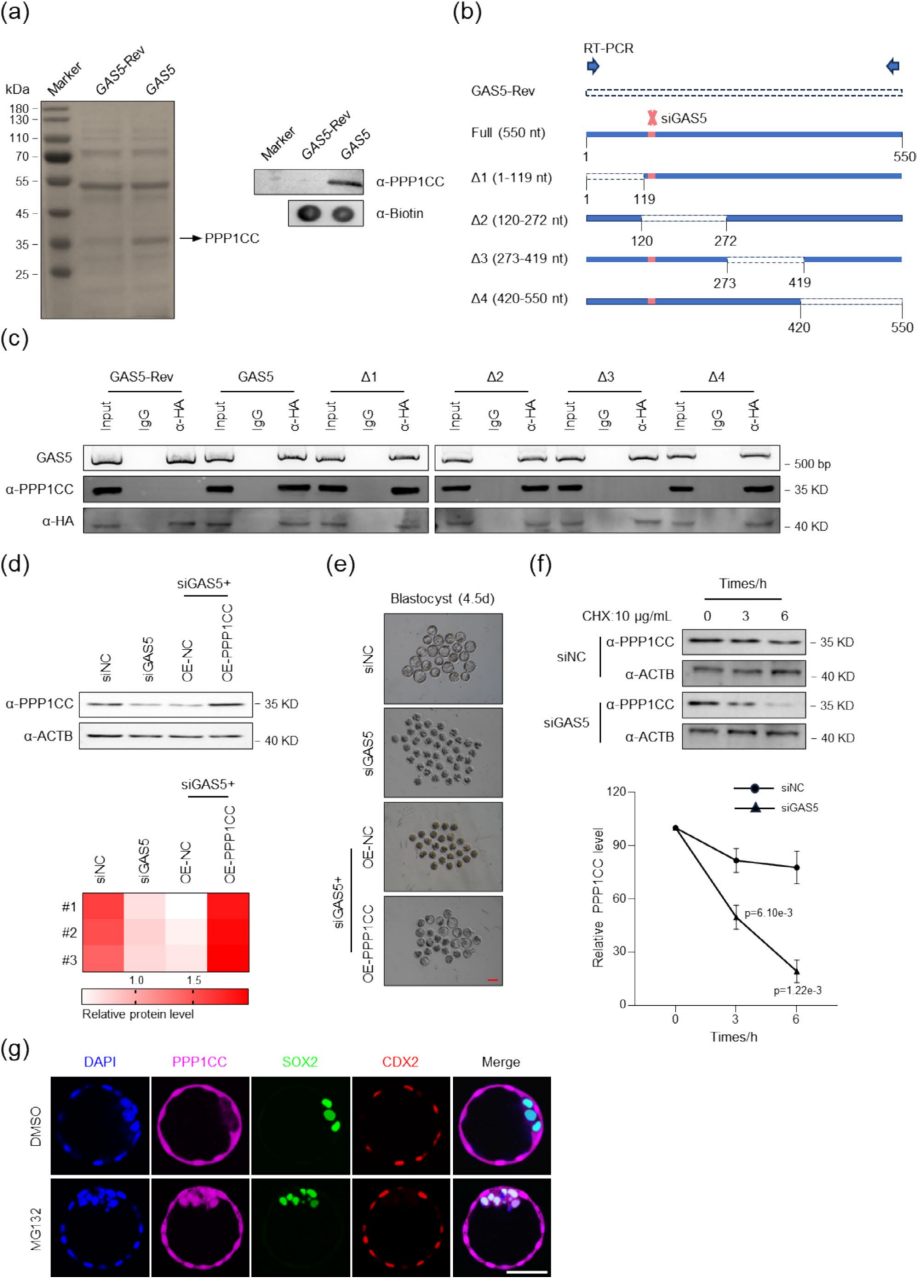
GAS5 regulates PPP1CC stability through direct binding. (a) GAS5 interacts with PPP1CC in vitro. Silver staining of SDS‐PAGE gel after RNA pull‐down shows proteins bound to GAS5 (right lane) versus GAS5‐Rev (reverse sequence control, middle lane). Assay performed using lysates from 5000 morula embryos. Western blot confirmation of PPP1CC binding to GAS5 from mass spectrometry results (RNA pull‐down followed by WB; α‐ anti‐). (b) Schematic of truncated GAS5 constructs used for functional rescue. GAS5‐Rev Full‐length reverse sequence control; siGAS5 Target site for GAS5 siRNA. Arrows indicate RT‐PCR primer locations for GAS5 or GAS5‐Rev detection. (c) Co‐immunoprecipitation (Co‐IP) in 293T cells using anti‐HA antibody (targeting HA‐tagged MS2 coat protein) confirms GAS5‐PPP1CC complex formation. *GAS5*‐Rev Antisense control RNA; α‐ anti‐. GAS5‐Rev primers were reverse‐orientation specific. Assays used ≈1 × 10^6^ cells per replicate. *n* = 3 biologically independent experiments. (d) Western blot analysis of PPP1CC protein levels in morula embryos co‐injected with GAS5 siRNA and *Ppp1cc* mRNA versus respective controls. Approximately 300 embryos per group were used across *n* = 3 biologically independent experiments. Heatmap shows quantified protein levels. α‐ anti‐. (e) Overexpression of PPP1CC rescued the embryonic developmental arrest phenotype caused by GAS5 deficiency. siRNA and mRNA was injected at 25 h phCG, and late blastocysts were imaged at 114 h phCG (4.5d). *n* = 3 biologically independent experiments. Scale bar 100 μm. (f) After the interference with GAS5 in embryo, the morula were treated with cycloheximide (CHX) (10 μg/mL) for 0, 3, and 6 h, respectively. Western blot was conducted to test the protein level of PPP1CC. Approximately 200 embryos per group were used across *n* = 3 biologically independent experiments. α‐, anti‐. (g) Immunofluorescence staining of PPP1CC, CDX2, and SOX2 at the blastocyst stage. Embryos were treated with MG132 (10 μM) after developing to the morula stage and then cultured until the blastocyst stage. Scale bar: 50 μm.

Moreover, RNA pull‐down assays and CRISPR‐Assisted RNA‐Protein Interaction Detection (CARPID) [33] confirmed the interaction between PPP1CC and GAS5 (Figure 5a and Figure S4c). To identify the key sequence within GAS5 involved in its regulatory mechanism, we generated four truncation mutants of *GAS5* (designated Δ1–Δ4) based on its exon distribution (Figure 5b). To avoid interference from the siGAS5 target sites within these constructs, we mutated the siGAS5 sites within each truncation. We also generated a full‐length GAS5 control with mutated siGAS5 sites (Full). GAS5‐Rev is the reverse sequence of full‐length *GAS5* (Figure 5b).

After that, we performed experiments in 293T cells. We used the MCP‐MS2 (MS2 bacteriophage coat protein) system to HA‐tag each fragment and performed co‐immunoprecipitation (Co‐IP) using an HA antibody. The results showed that Δ1, Δ2, and Δ4 could bind to PPP1CC, whereas Δ3 failed to bind (Figure 5c). Subsequent RT‐PCR analysis of the Co‐IP samples successfully detected the presence of the respective GAS5 fragments (Figure 5c). This indicates that GAS5 binds to PPP1CC and exerts its regulatory function through exons 5‐9 (corresponding to the Δ3 truncation region). Therefore, our findings demonstrate that GAS5 interacts with PPP1CC via its exons 5‐9 sequence to form an RNA‐protein complex, playing a crucial role in mouse early embryonic development.

### 3.7 GAS5 Stabilises PPP1CC in the TE

Collectively, these results demonstrate that while GAS5 exhibits exclusive TE localisation, PPP1CC transitions from uniform distribution in morula to TE‐specific targeting during blastocyst formation, ultimately enabling physical interaction between the two molecules. We thus propose a model wherein during the morula‐to‐blastocyst transition, PPP1CC undergoes degradation in inner cells due to GAS5 absence, whereas subcortically‐localised *GAS5* stabilises PPP1CC in outer cells, thereby driving lineage‐specific regulation. To further investigate whether subcortical‐localised GAS5 is the key for TE‐specific localisation of PPP1CC in blastocyst, we evaluate the stability of PPP1CC protein upon GAS5 depletion. The results showed that GAS5 depletion significantly reduced PPP1CC protein levels (Figure 5d). Moreover, overexpression of PPP1CC also rescued the embryonic developmental arrest and aberrant YAP phosphorylation caused by GAS5 dysregulation (Figure 5e and Figure S5a,b).

To investigate whether GAS5 regulates PPP1CC through protein stabilisation, we performed a protein stability assay. Control and GAS5‐knockdown embryos were cultured to the morula stage and then treated with cycloheximide (CHX) for 0, 3, and 6 h. Western blot analysis revealed that PPP1CC protein levels were lower in GAS5‐knockdown embryos compared to the control group at the 0‐h time point and decreased more rapidly with prolonged CHX treatment (Figure 5f). Based on these results, we hypothesised that the absence of GAS5 in the inner cells leads to PPP1CC degradation. To test this, morula‐stage embryos were treated with MG132, a proteasome inhibitor, with DMSO as a control, and then examined at the blastocyst stage by immunofluorescence staining. In MG132‐treated embryos, PPP1CC was detected in both inner and outer cells, whereas in control embryos, it remained predominantly localised to the outer cells (Figure 5g). These findings support the conclusion that the polarised distribution of GAS5 stabilises PPP1CC specifically in the outer cells of the blastocyst.

### 3.8 Knockdown of GAS5 Induces Developmental Arrest at the Morula Stage

These findings demonstrate that *GAS5* is indispensable for the TE‐specific localisation of PPP1CC, thus we propose that GAS5 serves as a pivotal regulator governing the first lineage differentiation. To that end, we interfered with GAS5 expression, which induced developmental arrest at the morula stage, indicating the essential role of GAS5 in ICM/TE segregation (Figure S6a–e). Co‐injection of full‐length GAS5 without LNA‐target site rescued the developmental phenotype, enabling blastocyst development in about 60% of embryos (Figure S6b–e). IF results revealed that the cytoplasmic accumulation of p‐YAP and diminished nuclear YAP in outer cells, revealing GAS5 knockdown recapitulated PPP1CC depletion phenotypes (Figure S6f–i).

Moreover, we overexpressed the aforementioned truncated fragments in embryos subjected to GAS5 knockdown. Developmental analysis revealed that injection of Δ1, Δ2, Δ4, or the Mut control could rescue the developmental arrest caused by GAS5 depletion, whereas injection of Δ3 or Rev. could not (Figure S7a–c). This further supports the reliability of the Co‐IP results. These findings confirmed that subcortical GAS5 governs TE‐specific PPP1CC stability, thereby directly driving Hippo‐YAP signalling during first lineage specification.

### 3.9 Exogenous GAS5 Biases Blastomeres Towards the TE Fate

**FIGURE 6:**
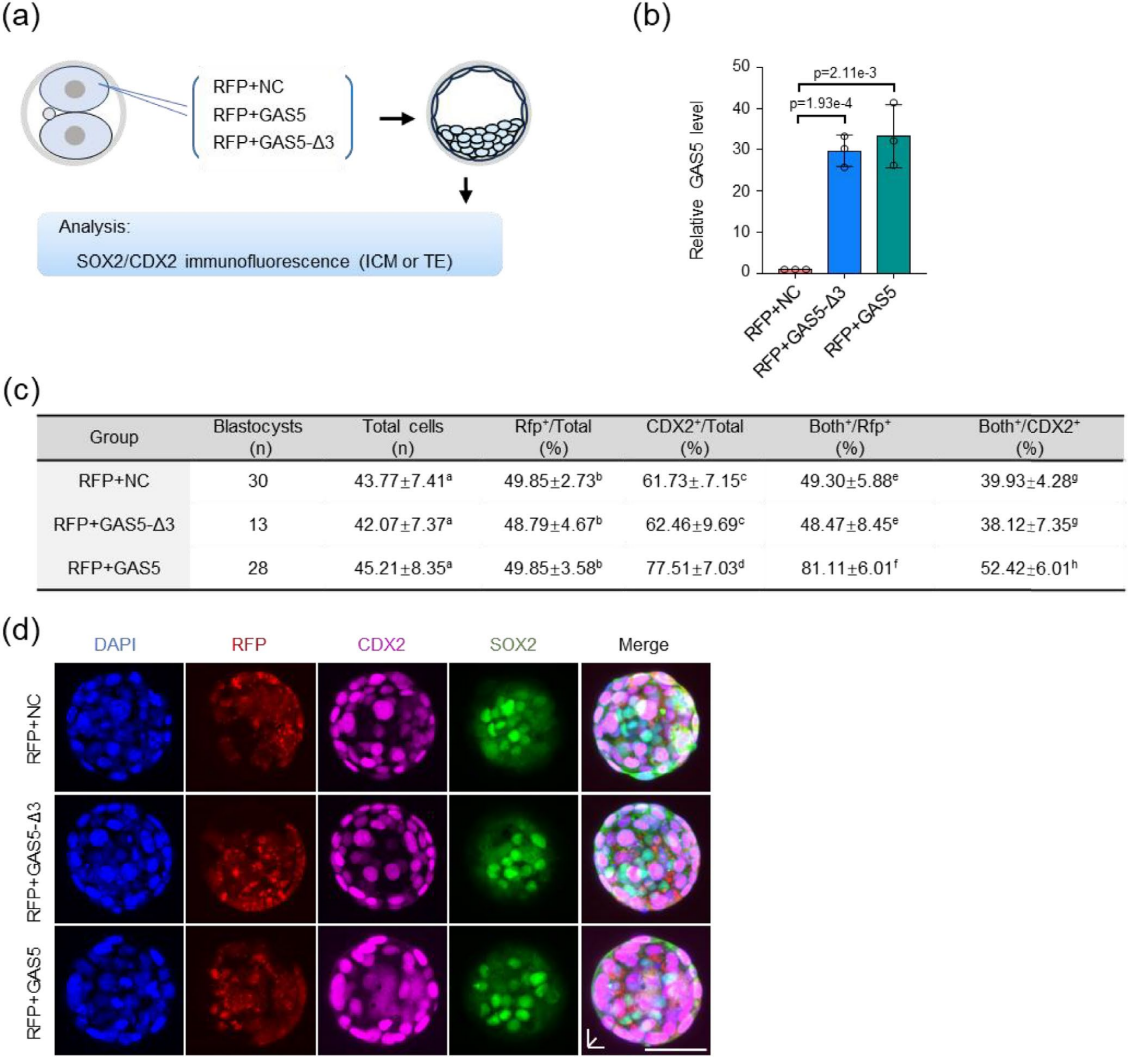
GAS5 ensures the TE fate. (a) Schematic overview. RFP + NC, RFP + GAS5 and RFP + GAS5‐Δ3 were injected into one blastomere of late two‐cell embryos, and the distributions of RFP positive cells were analysed at the blastocyst stage by CDX2 IF for the TE pattern. RFP, red fluorescent protein, NC, negative control. (b) qRT‐PCR analysis of relative GAS5 expression in embryos at eight‐cell stages. *n* = 3 biologically independent experiments. Two‐tailed Student's *t*‐test was used for the statistical analysis. The data are presented as the mean ± SEM. (c) Statistical data of the percentages of RFP‐ and CDX2‐positive cells among CDX2‐positive cells in RFP+NC, RFP+GAS5 and RFP + GAS5‐Δ3 groups. Both+denotes embryos positive for both CDX2 and RFP. Both+/RFP+ represents the percentage of RFP‐positive cdx2‐positive cells out of total RFP‐positive cells in the blastocyst, while Both+/cdx2+ indicates the percentage of RFP‐positive cdx2‐positive cells out of total cdx2‐positive cells. For statistical validity, each group was limited to blastocysts with 40%–60% RFP‐positive cells. Data are represented as mean ± SEM. Different letters within the same column indicate statistically significant differences (*p* < 0.001). (d) Fluorescent staining of blastocysts for CDX2, SOX2 and RFP, with CDX2 as a TE marker, SOX2 as an ICM marker and RFP as a lineage tracer. RFP + NC represents blastocysts developed after injecting RFP and NC into a single blastomere at the 2‐cell stage. RFP + GAS5 depicts blastocysts developed after injecting RFP and *GAS5*. RFP + GAS5‐Δ3 depicts blastocysts developed after injecting RFP and GAS5‐Δ3, differing in the number of daughter cells developed from the injected blastomere compared to the uninjected one. Scale bar 50 μm.

Given the critical role of GAS5 in the first lineage segregation, we next investigated whether GAS5 functions in early embryonic fate determination. We injected GAS5 into one blastomere of 2‐cell stage embryos, co‐injecting red fluorescent protein (RFP) mRNA as a lineage tracer. Embryos were then monitored until the blastocyst stage. CDX2 and SOX2 served as markers for TE and ICM, respectively (Figure 6a). We confirmed elevated levels of GAS5 by qRT‐PCR at the eight‐cell stage following overexpression (Figure 6b).

We analysed the RFP‐positive cell population in blastocysts derived from the three groups (RFP + NC, RFP + GAS5 and RFP + GAS5‐Δ3). Total blastocyst cell numbers and the percentage of RFP‐positive cells were similar across groups. Evaluation of CDX2 expression revealed a significantly higher percentage of CDX2‐positive cells in the entire blastocyst in the RFP + GAS5 group (77.51% ± 7.03%) compared to the RFP + NC group (61.73% ± 7.15%) and RFP + GAS5‐Δ3 group (62.46% ± 9.69%). Crucially, the percentage of CDX2‐positive cells among the RFP‐positive cells was notably higher in the GAS5 + RFP group (81.11% ± 6.01%) compared to the RFP + NC group (49.30% ± 5.88%) and RFP + GAS5‐Δ3 group (48.47% ± 8.45%). Similarly, the proportion of RFP‐positive cells located within the TE compartment was higher in the GAS5 + RFP group (52.42% ± 6.01%) than in the RFP + NC group (39.93% ± 4.28%) and RFP + GAS5‐Δ3 group (38.12% ± 7.35%) (Figure 6c,d). These results indicate that the function of GAS5 is dependent on its binding to PPP1CC, rather than a non‐specific effect of lncRNA overexpression.

## 4 Discussion

**FIGURE 7:**
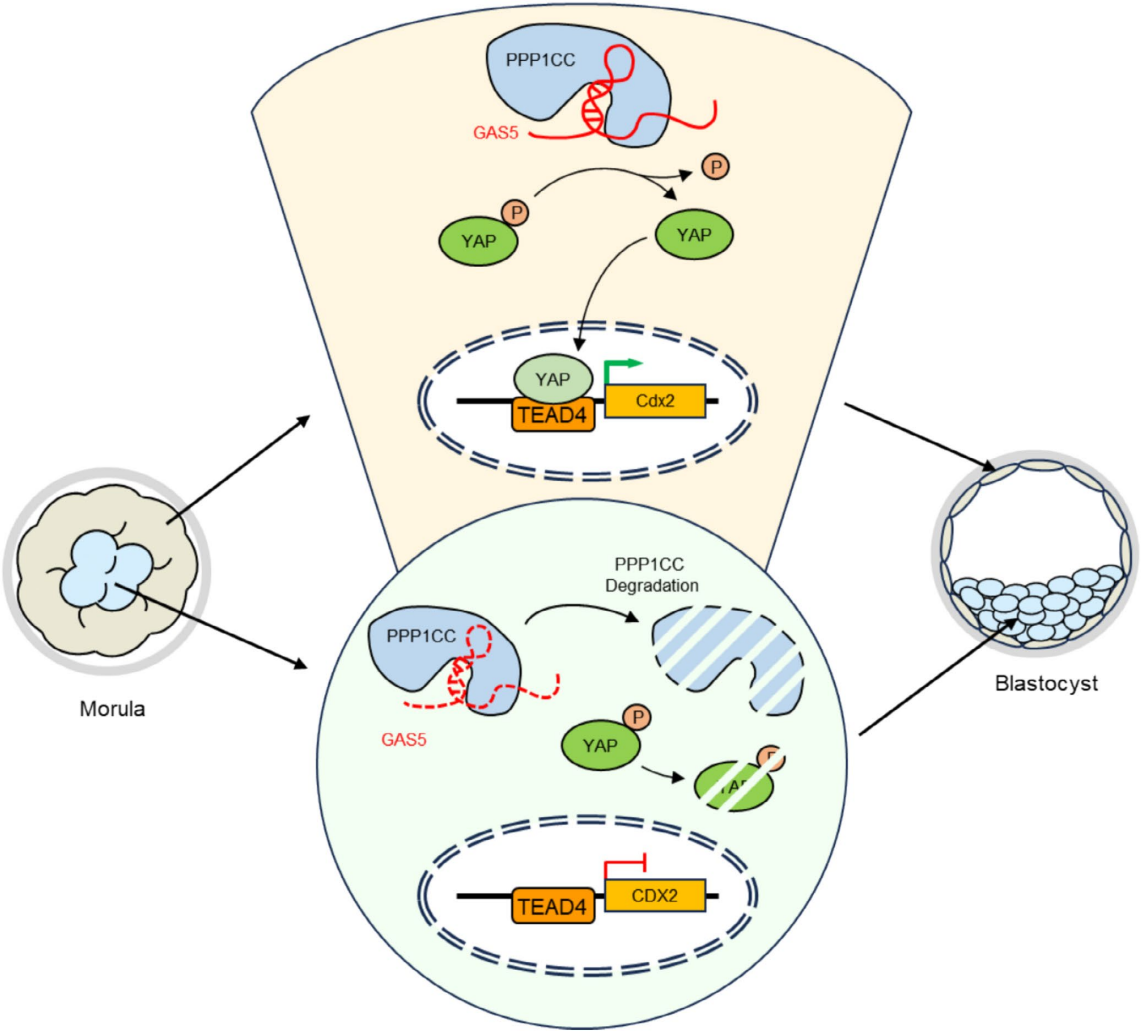
Schematic model for the PPP1CC‐regulated Hippo‐YAP signalling.

The Hippo‐YAP signalling pathway is a key regulator of the first lineage specification in mouse preimplantation embryos [8]. However, the mechanism enabling TE‐specific YAP activation remains elusive. Existing studies on Hippo‐YAP pathway regulation concentrate overwhelmingly on kinases, largely neglecting how phosphatases control YAP during early embryonic development in mice [1, 9, 10]. In this study, we demonstrated that the specific localisation of phosphatase PPP1CC in the TE of mouse blastocysts is achieved through GAS5, which is subcortically localised. Mechanistically, GAS5 stabilises PPP1CC in TE, promoting YAP dephosphorylation and nuclear translocation in blastocyst, which drives TE‐specific gene expression and enables proper blastocyst development (Figure 7). Our delineation of the GAS5‐PPP1CC‐YAP axis in mouse preimplantation embryos offers novel mechanistic perspectives for understanding embryogenesis and lineage specification.

Phosphatase‐mediated regulation of the Hippo‐YAP pathway has been previously reported, including the STRIPAK‐PP2A complex targeting Hippo/MST kinases [34, 35, 36]; PP1 (a member of the PPP superfamily) directly inducing YAP dephosphorylation via ASPP2‐linked apical‐basal polarity complexes [37, 38]; PTPN14 negatively regulating YAP through WW domain interactions [39] and PPM1A binding to eliminate YAP S127 phosphorylation [18]. We here identified PPP1CC as the phosphatase regulating Hippo‐YAP signalling during first lineage specification in mouse early embryos. Moreover, while we have confirmed the role of PPP1CC in regulating lineage differentiation, given its progressively increasing expression levels from the 2‐cell to morula stage, we hypothesise that PPP1CC may also play an important role in zygotic genome activation (ZGA). This potential function warrants further investigation. Furthermore, the dual localisation of PPP1CC in both cytoplasm and nucleus suggests potential functional significance in both compartments. In future work, we will investigate the nuclear‐specific functions of PPP1CC during embryonic development.

Additionally, we note that another phosphatase, PPP2CB, is also highly expressed in early embryos (Figure 1a). Studies in mouse oocytes indicate PPP2CB upregulation drives meiotic arrest phenotypes [40], while paradoxically, PPP2CB‐knockout mice exhibit normal behaviour and retained fertility [41]. The pronounced expression of phosphatase PPP2CB at the 2‐cell embryo stage prompts investigation into its putative functions in dephosphorylating critical signalling effectors beyond known pathways. Whether PPP2CB exhibits functional redundancy or synergy with PPP1CC remains a subject worthy of further investigation.

Excitingly, we discovered that TE‐specific localisation of PPP1CC during the blastocyst stage is regulated by lncRNA GAS5, which persistently localises to the subcortical region throughout mouse early embryonic development. However, the mechanism by which GAS5 regulates PPP1CC stability remains incompletely understood. We postulate that in inner cells, the absence of GAS5 leads to PPP1CC degradation. This hypothesis awaits experimental validation.

Moreover, the subcortical localisation of GAS5 may be associated with the subcortical maternal complex (SCMC). SCMC—an oocyte‐to‐early embryo‐specific protein complex—is essential for mouse embryogenesis [42, 43]. Integrity of the SCMC is contingent upon a tripartite module comprising MATER, TLE6 and FLOPED. Disruption of any core component significantly reduces the abundance of other constituents and results in developmental arrest at the 2‐ to 4‐cell stage [44, 45, 46]. Furthermore, our mass spectrometry analysis of GAS5 pull‐down assays identified components of the subcortical maternal complex (SCMC), including OOEP (FLOPED), TLE6 and PADI6 (Table S1). Whether GAS5, a key determinant of TE lineage fate at the 2‐cell stage, functionally interacts with the SCMC, and whether SCMC contributes to the subcortical positioning of GAS5, remain critical open questions requiring further investigation.

## Author Contributions

Jianwu Wang, Jiaqiang Wang conceived and designed the study. Jianwu Wang, Yan Zhang, Laijin Wu, Hongshuang Xie, Guang Yang, Yiwei Zhang, Cheng Huang, Shanyi Chou, Xuehan Li performed the experiments and analysed the data. Jianwu Wang wrote the manuscript. Zhonghua Liu and Jiaqiang Wang supervised the project.

## Conflicts of Interest

The authors declare no conflicts of interest.

## Supporting information


**Figure S1:** Phosphatase PPP1CC promotes YAP transcriptional activity. (a) Schematic diagram illustrating the detection principle of the dual‐luciferase reporter system. TF, Transcription factors. WT, wild type. MT, mutant. (b) Protein intensities were normalised. *n* = 3 biologically independent experiments. Two‐tailed Student's *t*‐test was used for the statistical analysis. The data are presented as the mean ± SEM. (c) Western blot analysis was employed to assess the p‐YAP, YAP, p‐LATS1, LATS1 protein level. Protein intensities were normalised. Phosphorylation levels are represented as the ratio of phosphorylated protein to total protein. *n* = 3 biologically independent experiments. Two‐tailed Student's *t*‐test was used for the statistical analysis. The data are presented as the mean ± SEM. (d) Protein intensities were normalised. Phosphorylation levels are represented as the ratio of phosphorylated protein to total protein. *n* = 3 biologically independent experiments. Two‐tailed Student's *t*‐test was used for the statistical analysis. The data are presented as the mean ± SEM. n.s., not significant. (e) Protein intensities were normalised. Phosphorylation levels are represented as the ratio of phosphorylated protein to total protein. *n* = 3 biologically independent experiments. Two‐tailed Student's *t*‐test was used for the statistical analysis. The data are presented as the mean ± SEM.
**Figure S2:** Verification of PPP1CC knockdown efficiency in preimplantation embryos. (a) Effective PPP1CC mRNA knockdown mediated by RNAi. siRNA was injected at 25 h post‐hCG (phCG), and embryos were collected at phCG 62 h (4‐cell stage), 74 h (8‐cell stage), 90 h (morula stage), and 114 h (blastocyst stage) for qRT‐PCR analysis. Approximately 100 embryos per developmental stage were analysed across *n* = 3 biologically independent experiments. Two‐tailed Student's *t*‐test was used for the statistical analysis. The data are presented as the mean ± SEM. (b) Western blot analysis was employed to assess the PPP1CC protein level. The morula stage embryos (300 embryos per group) were used for this analysis, and three replicates were performed, yielding consistent results. α‐ACTB was utilised as the loading control to normalise the protein levels. α‐, anti‐. (c) Immunofluorescence staining analysis was conducted on morula embryos using a PPP1CC antibody. Representative images were captured from three independent experiments. In the left panel, merged images display the colocalization of PPP1CC (green) and DNA (blue). Control (*n* = 11); PPP1CC‐si1 (*n* = 10); PPP1CC‐si2 (*n* = 13). ‘*n*’ denotes the number of embryos per group. Scale bar 50 μm. The right panel shows the relative intensity of PPP1CC signal compared to control embryos. Two‐tailed Student's *t*‐test was used for the statistical analysis. The data are presented as the mean ± SEM. (d) Embryonic morphology at preimplantation stages following PPP1CC knockdown. Scale bar 100 μm. (e) Embryonic development following PPP1CC mRNA injection. MO morula; BL blastocyst. PPP1CC mRNA concentration for both control and overexpression groups was 150 ng/μL.
**Figure S3:** PPP1CC overexpression leads to an increase in TE.
(a) Western blot analysis was employed to assess the PPP1CC protein level. The morula stage embryos (300 embryos per group) were used for this analysis, and three replicates were performed, yielding consistent results. α‐ACTB was utilised as the loading control to normalise the protein levels. α‐, anti‐. (b)PPP1CC overexpression leads to an increase in the number of CDX2‐positive cells. Quantitative analysis of CDX2‐positive cell numbers in Figure 3a,c. The data are presented as the mean ± SEM. (c) Immunofluorescence staining of CDX2 and SOX2 at the blastocyst stage. Images are representative of three independent experiments (Control *n* = 12; PPP1CC‐oe *n* = 10). ‘*n*’ denotes the number of embryos per group. Scale bar 50 μm. (d) Dot plots displaying the average counts of total cells, TE cells, and ICM cells per blastocyst embryo in control and ppp1cc‐oe embryos at 4.5dpc. *n* = 3 biological replicates. TE, trophectoderm. ICM, inner cell mass. The number of blastocysts in the control group and the ppp1cc‐oe group were 12 and 10, respectively. Data are presented as mean values ± SEM and were analysed using two‐tailed unpaired *t*‐tests.
**Figure S4:** GAS5 localises to the subcortical region and interacts with PPP1CC. (a) Subcellular localisation analysis of GAS5 by RNA fractionation and RT‐qPCR analysis. The results show that GAS5 locate in the cytoplasm. The error bars represent SEM. Cyt, cytoplasm; Nuc, nucleoplasm. Gapdh and U6 act as cyt and nuc control, respectively. About 200 8‐cell embryos were used for each experiment, and three experimental replicates were performed. The data are presented as the mean ± SEM. (b) qRT‐PCR analysis of relative *Ppp1cc* expression across developmental stages. Data from three independent biological replicates normalised to *Hprt*. Box plots show medians (center lines) and interquartile ranges (box edges). (c) Schematic diagram of CARPID. The specific sgRNA‐guided CRISPR/dCasRx recognises the GAS5 single‐stranded region. BASU fused to CRISPR/dCasRx adds biotin to adjacent binding proteins. Streptavidin coated beads are used to purify the binding proteins. RBP, RNA binding proteins. GAS5 interacting proreins analysed by CARPID in blastocysts (right lane). Pronuclear injection of vectors was performed at the zygote stage, followed by doxycycline (Dox)‐inducible expression initiation at the morula stage. A total of 530 blastocysts were collected at the blastocyst stage. Western blotting detection of PPP1CC in input and streptavidin pulldown samples of control (GAS5‐Rev, GAS5 reverse sequence) and GAS5 sgRNA (GAS5). CRISPR/dCasRx‐V5 (V5‐Tag) and α‐ACTB serves as a positive and negative control, respectively. α‐, anti‐.
**Figure S5:** PPP1CC overexpression rescues the defects caused by GAS5 knockdown. (a) Embryonic development after microinjection. Different letters in same column indicate significant difference (*p* < 0.001). (b) Immunofluorescence staining images of p‐YAP and quantitative analysis of fluorescence density across the four groups. Images are representative of three independent experiments. Scale bar 50 μm.
**Figure S6:** GAS5 knockdown results in embryonic arrest at the morula stage. (a) Locked nucleic acid (LNA) efficiently mediated GAS5 knockdown. Design two LNA‐modified siRNA fragments. LNA was injected at phCG 25 h, and embryos were collected at phCG 48 h at late two‐cell stage for RT‐qPCR analysis. The control group represents the LNA‐modified siRNA control fragments. *n* = 3 biologically independent experiments. Two‐tailed Student's *t*‐test was used for the statistical analysis. The data are presented as the mean ± SEM. (b) GAS5 overexpression assay. Full‐length GAS5 RNA was injected at 25 h post‐hCG (phCG) to induce overexpression, and embryos were collected at phCG 48 h at late two‐cell stage for qRT‐PCR analysis. *n* = 3 biologically independent experiments. Two‐tailed Student's *t*‐test was used for the statistical analysis. The data are presented as the mean ± SEM. (c) Statistical Analysis of Developmental Rates upon GAS5 Knockdown and Overexpression. Bar plots show the developmental rates of the control group, knockdown group and overexpression group at the blastocyst stage (E4.5). *n* = 3 experimental replicates. (d) Embryonic morphology at preimplantation stages following GAS5 knockdown and overexpression. Scale bar 100 μm. (e) Embryonic development after microinjection. Different letters in same column indicate significant difference (*p* < 0.001). (f) Representative immunofluorescence images of p‐YAP in control, GAS5 knockdown (siGAS5), and GAS5 overexpressing (GAS5‐OE) morula. Images are representative of three independent experiments. Scale bar 50 μm. (g) Quantification of fluorescence intensity. Morula were divided into outer and inner cells. p‐YAP intensity in the cytoplasm (Cyt) and nucleoplasm (Nuc) was quantified separately for outer and inner cells. Two‐tailed Student's *t*‐test was used for the statistical analysis. The data are presented as the mean ± SEM. (h) Representative immunofluorescence images of YAP in control, GAS5 knockdown (siGAS5), and GAS5 overexpressing (GAS5‐OE) morula. Images are representative of three independent experiments. Scale bar 50 μm. (i) Quantification of fluorescence intensity. YAP intensity in the cytoplasm (Cyt) and nucleoplasm (Nuc) was quantified separately for outer and inner cells of morula. Two‐tailed Student's *t*‐test was used for the statistical analysis. The data are presented as the mean ± SEM.
**Figure S7:** Functional rescue of GAS5 depletion requires specific RNA domains. (a) Blastocyst rates upon overexpression of six GAS5 truncation mutants during GAS5 depletion. siGAS5 and siGAS5 + Δ3 significantly reduced blastocyst formation. *n* = 3 biologically independent experiments. Two‐tailed Student's *t*‐test was used for the statistical analysis. The data are presented as the mean ± SEM. (b) Embryonic development after microinjection. Different letters in same column indicate significant difference (*p* < 0.001). *n* = 3 biologically independent experiments. (c) Morphological phenotypes of embryos overexpressing truncated GAS5 RNAs under GAS5 depletion. Embryos were imaged at 110 h phCG (blastocyst stage). siGAS5 + Δ1/Δ2/Δ4/Mut groups developed to blastocysts, while siGAS5 and siGAS5 + Δ3 arrested at morula stage. Scale bar 100 μm.

**Table S1:** cpr70155‐sup‐0002‐TableS1.xlsx.

## Data Availability

The sequencing data of RIP‐seq has been deposited in the Genome Sequence Archive of Beijing Institute of Genomics, Chinese Academy of Sciences (GSA, http://gsa.big.ac.cn/) with the accession number of CRA057793.
