# Supplementary material for: Phosphatase PPP1CC Regulates the First Lineage Segregation by GAS5 in Mouse Preimplantation Embryos
Source: Cell Prolif. 2025 Dec 16;59(6):e70155. doi: 10.1111/cpr.70155 (PMC13241819; doi:10.1111/cpr.70155)
Supplement: Supplementary file 1 — Figure S1: Phosphatase PPP1CC promotes YAP transcriptional activity. (a) Schematic diagram illustrating the detection principle of the dual‐luciferase reporter system. TF, Transcription factors. WT, wild type. MT, mutant. (b) Protein intensities were normalised. n = 3 biologically independent experiments. Two‐tailed Student's t‐test was used for the statistical analysis. The data are presented as the mean ± SEM. (c) Western blot analysis was employed to assess the p‐YAP, YAP, p‐LATS1, LATS1 protein level. Protein intensities were normalised. Phosphorylation levels are represented as the ratio of phosphorylated protein to total protein. n = 3 biologically independent experiments. Two‐tailed Student's t‐test was used for the statistical analysis. The data are presented as the mean ± SEM. (d) Protein intensities were normalised. Phosphorylation levels are represented as the ratio of phosphorylated protein to total protein. n = 3 biologically independent experiments. Two‐tailed Student's t‐test was used for the statistical analysis. The data are presented as the mean ± SEM. n.s., not significant. (e) Protein intensities were normalised. Phosphorylation levels are represented as the ratio of phosphorylated protein to total protein. n = 3 biologically independent experiments. Two‐tailed Student's t‐test was used for the statistical analysis. The data are presented as the mean ± SEM. Figure S2: Verification of PPP1CC knockdown efficiency in preimplantation embryos. (a) Effective PPP1CC mRNA knockdown mediated by RNAi. siRNA was injected at 25 h post‐hCG (phCG), and embryos were collected at phCG 62 h (4‐cell stage), 74 h (8‐cell stage), 90 h (morula stage), and 114 h (blastocyst stage) for qRT‐PCR analysis. Approximately 100 embryos per developmental stage were analysed across n = 3 biologically independent experiments. Two‐tailed Student's t‐test was used for the statistical analysis. The data are presented as the mean ± SEM. (b) Western blot analysis was employed to asse [file CPR-59-e70155-s001.docx]

**Phosphatase PPP1CC regulates** **the first** **lineage segregation by GAS5 in mouse preimplantation embryos**

Jianwu Wang^1^, Yan Zhang^1^, Laijin Wu^1^, Hongshuang Xie^1^, Guang Yang^1^, Yiwei Zhang^1^, Cheng Huang^1^, Shanyi Chou^1^, Xuehan Li^1^, Zhonghua Liu^1#^, Jiaqiang Wang^1#^

Affiliations

^1^ Key Laboratory of Animal Cellular and Genetics Engineering of Heilongjiang Province, College of Life Science, Northeast Agricultural University, Harbin, 150030, P. R. China.

^#^ Correspondence and requests for materials should be addressed to

Zhonghua Liu (liuzhonghua@neau.edu.cn)

Jiaqiang Wang (wangjiaqiang@neau.edu.cn)

**Supplemental Data**


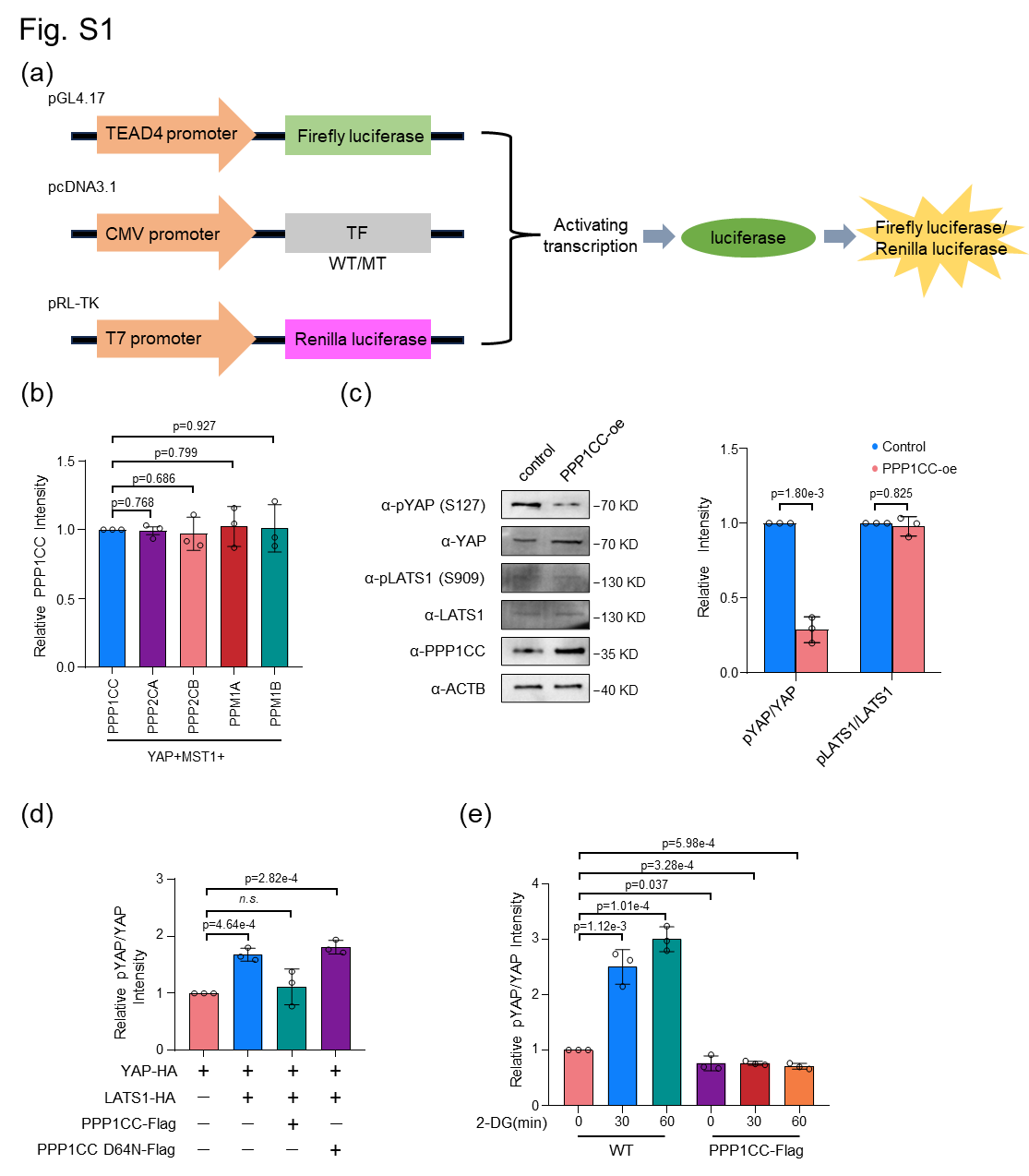


**Fig. S1 Phosphatase PPP1CC promotes YAP transcriptional activity.**
**(a**) schematic diagram illustrating the detection principle of the dual-luciferase reporter system. TF, Transcription factors. WT, wild type. MT, Mutant.

**(b**) Protein intensities were normalized. *n* = 3 biologically independent experiments. Two-tailed Student’s t-test was used for the statistical analysis. The data are presented as the mean ± SEM.

**(c**) Western blot analysis was employed to assess the p-YAP, YAP, p-LATS1, LATS1 protein level. Protein intensities were normalized. Phosphorylation levels are represented as the ratio of phosphorylated protein to total protein. *n* = 3 biologically independent experiments. Two-tailed Student’s t-test was used for the statistical analysis. The data are presented as the mean ± SEM.

**(d**) Protein intensities were normalized. Phosphorylation levels are represented as the ratio of phosphorylated protein to total protein. *n* = 3 biologically independent experiments. Two-tailed Student’s t-test was used for the statistical analysis. The data are presented as the mean ± SEM. n.s., not significant.

**(e**) Protein intensities were normalized. Phosphorylation levels are represented as the ratio of phosphorylated protein to total protein. *n* = 3 biologically independent experiments. Two-tailed Student’s t-test was used for the statistical analysis. The data are presented as the mean ± SEM.


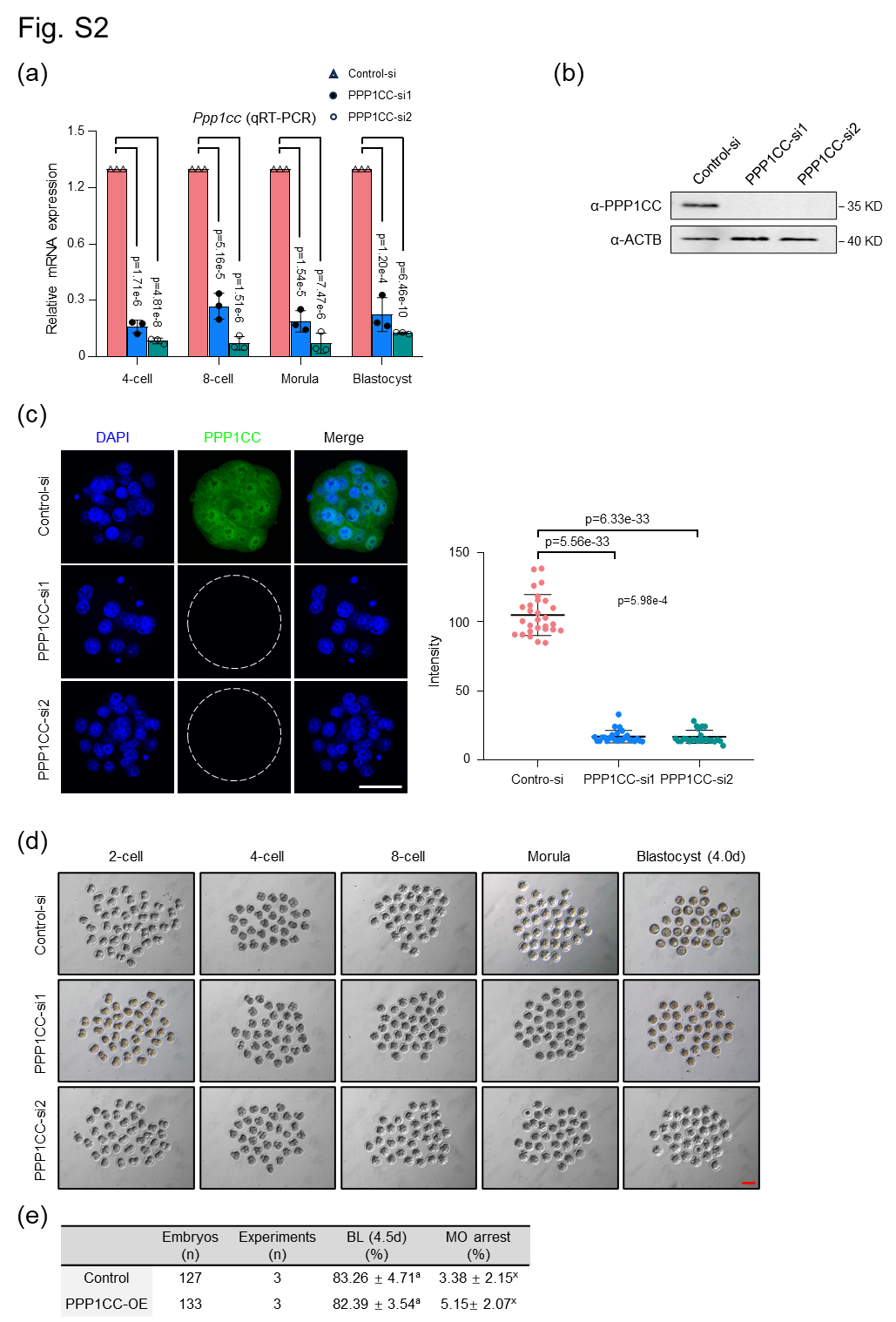


**Fig. S2 Verification of PPP1CC knockdown efficiency in preimplantation embryos.**

**(a**) Effective PPP1CC mRNA knockdown mediated by RNAi. siRNA was injected at 25 h post-hCG (phCG), and embryos were collected at phCG 62 h (4-cell stage), 74 h (8-cell stage), 90 h (morula stage), and 114 h (blastocyst stage) for qRT-PCR analysis. Approximately 100 embryos per developmental stage were analyzed across *n* = 3 biologically independent experiments. Two-tailed Student’s t-test was used for the statistical analysis. The data are presented as the mean ± SEM.

**(b**) Western blot analysis was employed to assess the PPP1CC protein level. The morula stage embryos (300 embryos per group) were used for this analysis, and three replicates were performed, yielding consistent results. α-ACTB was utilized as the loading control to normalize the protein levels. α-, anti-.

**(c**) Immunofluorescence staining analysis was conducted on morula embryos using a PPP1CC antibody. Representative images were captured from three independent experiments. In the left panel, merged images display the colocalization of PPP1CC (green) and DNA (blue). Control (*n* = 11); PPP1CC-si1(*n* = 10); PPP1CC-si2(*n* = 13). "n" denotes the number of embryos per group. Scale bar 50 μm. The right panel shows the relative intensity of PPP1CC signal compared to control embryos. Two-tailed Student’s t-test was used for the statistical analysis. The data are presented as the mean ± SEM.

**(d**) Embryonic morphology at preimplantation stages following PPP1CC knockdown. Scale bar 100 μm.

**(e**) Embryonic development following PPP1CC mRNA injection. MO Morula; BL Blastocyst. PPP1CC mRNA concentration for both control and overexpression groups was 150 ng/μL.


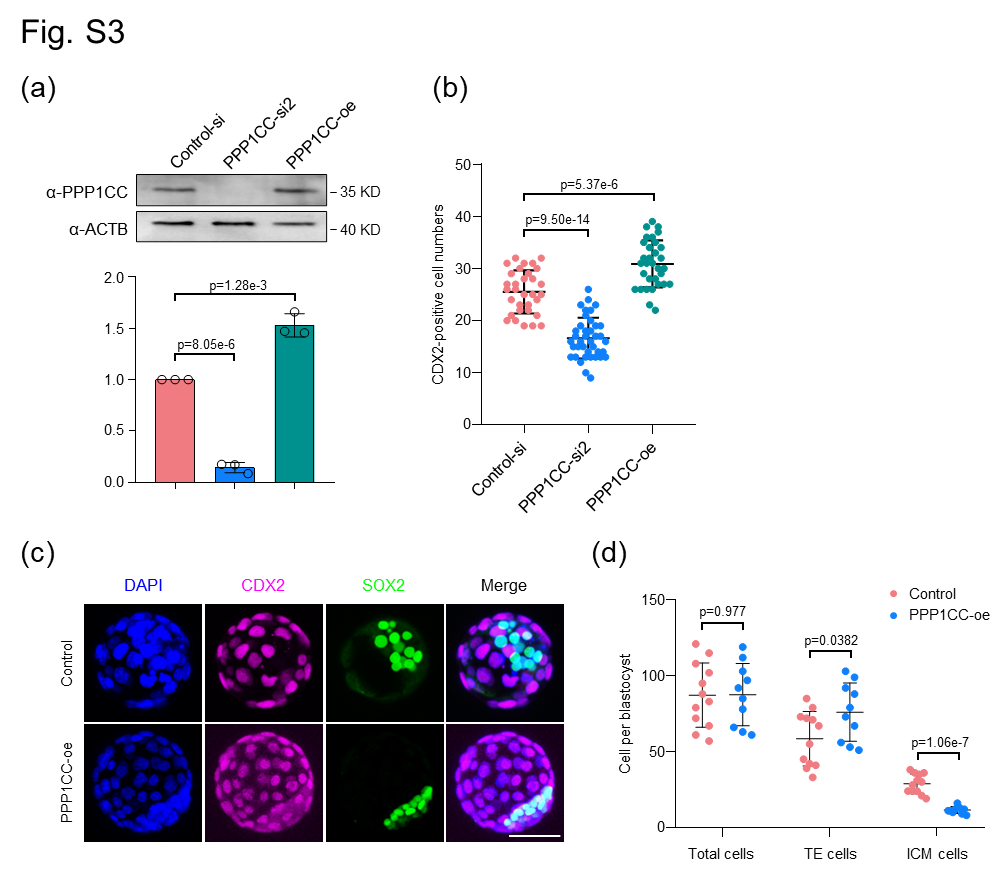


**Fig. S3 PPP1CC overexpression leads to an increase in TE.**

**(a**) Western blot analysis was employed to assess the PPP1CC protein level. The morula stage embryos (300 embryos per group) were used for this analysis, and three replicates were performed, yielding consistent results. α-ACTB was utilized as the loading control to normalize the protein levels. α-, anti-.

**(b**)PPP1CC overexpression leads to an increase in the number of CDX2-positive cells. Quantitative analysis of CDX2-positive cell numbers in Fig. 3a and 3c. The data are presented as the mean ± SEM.

**(c**) Immunofluorescence staining of CDX2 and SOX2 at the blastocyst stage. Images are representative of three independent experiments (Control n = 12; PPP1CC-oe n = 10). "n" denotes the number of embryos per group. Scale bar 50 μm.

**(d**) Dot plots displaying the average counts of total cells, TE cells, and ICM cells per blastocyst embryo in control and ppp1cc-oe embryos at 4.5dpc. n = 3 biological replicates. TE, trophectoderm. ICM, inner cell mass. The number of blastocysts in the control group and the ppp1cc-oe group were 12 and 10, respectively. Data are presented as mean values ± SEM and were analyzed using two-tailed unpaired t-tests.


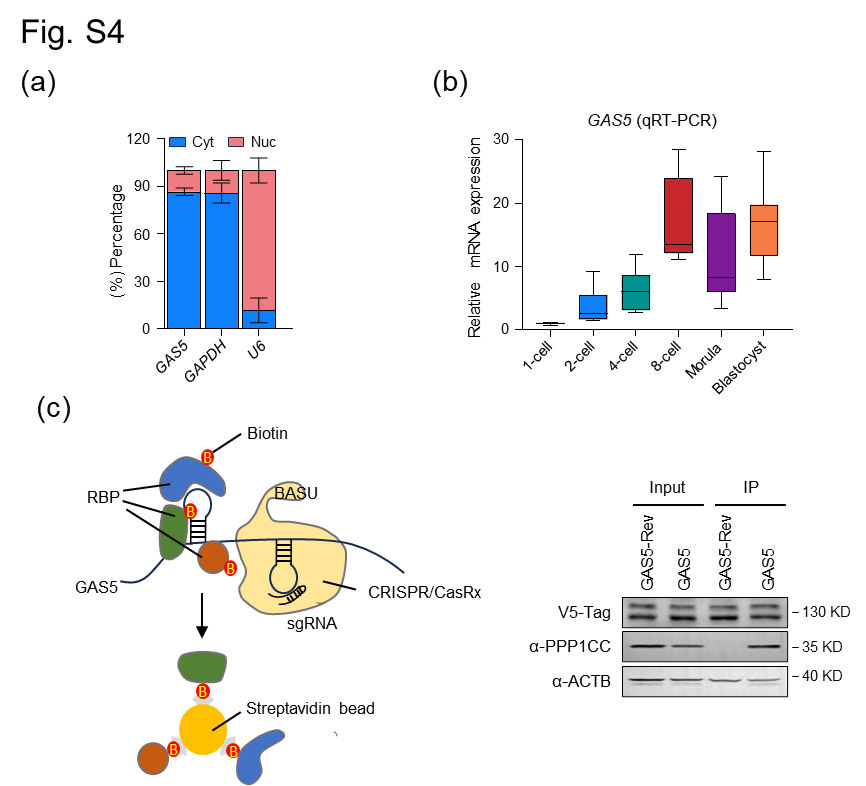


**Fig. S4 GAS5 localizes to the subcortical region and interacts with PPP1CC.**

**(a**) Subcellular localization analysis of GAS5 by RNA fractionation and RT-qPCR analysis. The results show that GAS5 locate in the cytoplasm. The error bars represent SEM. Cyt, cytoplasm; Nuc, nucleoplasm. Gapdh and U6 act as cyt and nuc control, respectively. About 200 8-cell embryos were used for each experiment, and three experimental replicates were performed. The data are presented as the mean ± SEM.

**(b**) qRT-PCR analysis of relative *Ppp1cc* expression across developmental stages. Data from three independent biological replicates normalized to *Hprt*. Box plots show medians (center lines) and interquartile ranges (box edges).
**(c**) Schematic diagram of CARPID. The specific sgRNA-guided CRISPR/dCasRx recognizes the GAS5 single-stranded region. BASU fused to CRISPR/dCasRx adds biotin to adjacent binding proteins. Streptavidin coated beads are used to purify the binding proteins. RBP, RNA binding proteins. GAS5 interacting proreins analyzed by CARPID in blastocysts (right lane). Pronuclear injection of vectors was performed at the zygote stage, followed by doxycycline (Dox)-inducible expression initiation at the morula stage. A total of 530 blastocysts were collected at the blastocyst stage. Western blotting detection of PPP1CC in input and streptavidin pulldown samples of control (GAS5-Rev, GAS5 reverse sequence) and GAS5 sgRNA (GAS5). CRISPR/dCasRx-V5(V5-Tag) and α-ACTB serves as a positive and negative control,respectively. α-, anti-.


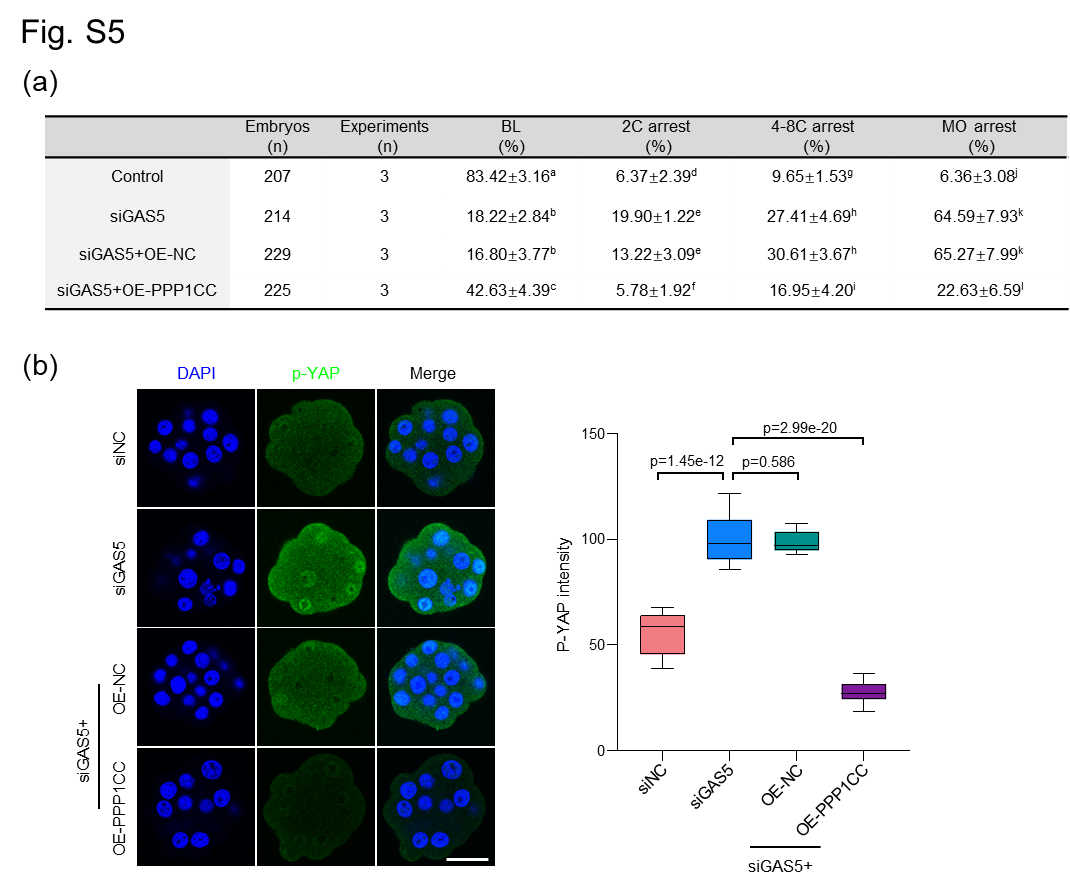


**Fig. S5 PPP1CC overexpression rescues the defects caused by GAS5 knockdown.**

**(a**) Embryonic development after microinjection. Different letters in same column indicate significant difference (P < 0.001).

**(b**) Immunofluorescence staining images of p-YAP and quantitative analysis of fluorescence density across the four groups. Images are representative of three independent experiments. Scale bar 50 μm.


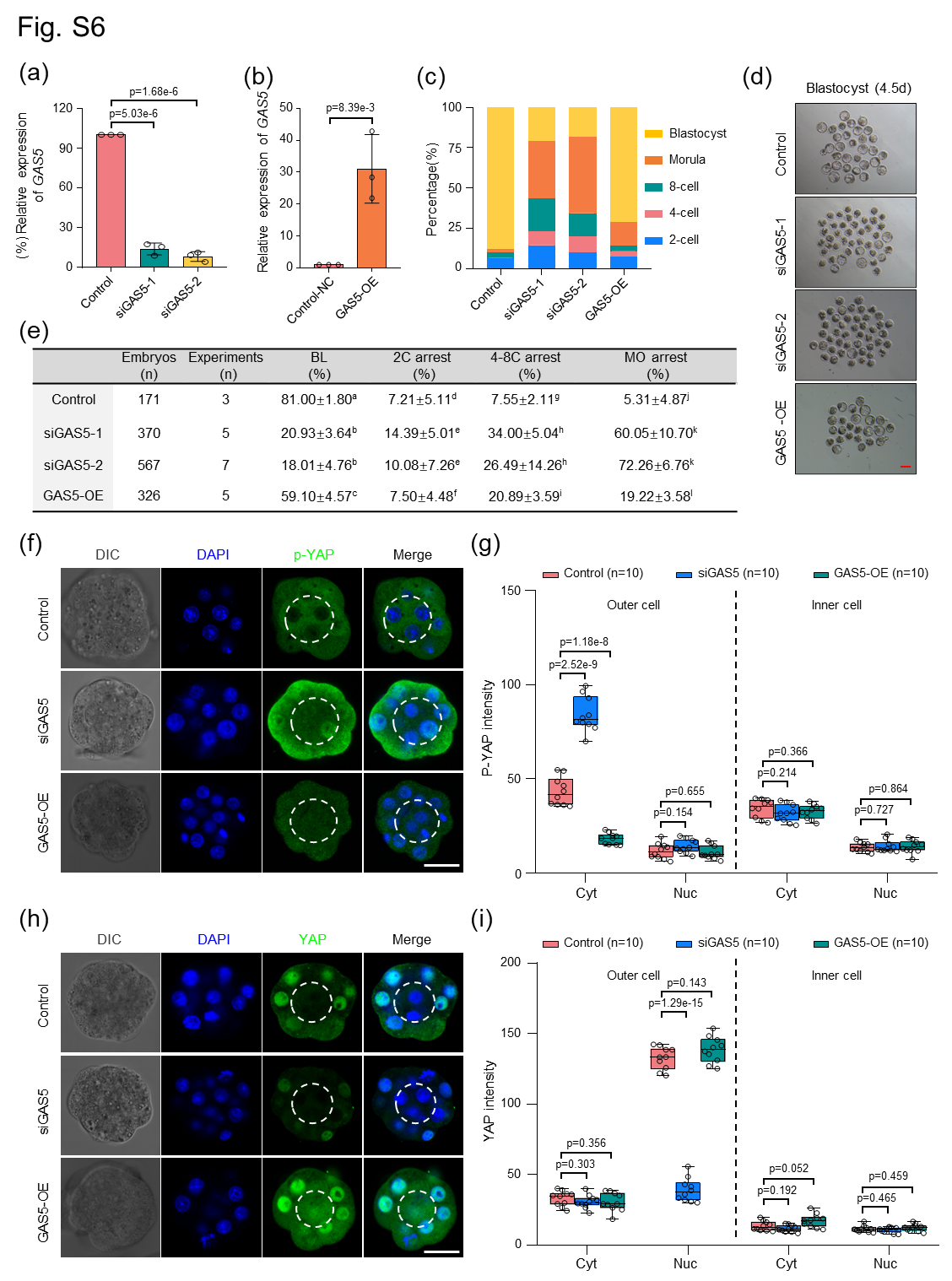


**Fig. S6 GAS5 knockdown results in embryonic arrest at the morula stage**

**(a**) Locked nucleic acid (LNA) efficiently mediated GAS5 knockdown. Design two LNA-modified siRNA fragments. LNA was injected at phCG 25 h, and embryos were collected at phCG 48 h at late two-cell stage for RT-qPCR analysis. The control group represents the LNA-modified siRNA control fragments. *n* = 3 biologically independent experiments. Two-tailed Student’s t-test was used for the statistical analysis. The data are presented as the mean ± SEM.

**(b**) GAS5 overexpression assay. Full-length GAS5 RNA was injected at 25 h post-hCG (phCG) to induce overexpression, and embryos were collected at phCG 48 h at late two-cell stage for qRT-PCR analysis. *n* = 3 biologically independent experiments. Two-tailed Student’s t-test was used for the statistical analysis. The data are presented as the mean ± SEM.

**(c**) Statistical Analysis of Developmental Rates upon GAS5 Knockdown and Overexpression. Bar plots show the developmental rates of the control group, knockdown group and overexpression group at the blastocyst stage (E4.5). n = 3 experimental replicates.

**(d**) Embryonic morphology at preimplantation stages following GAS5 knockdown and overexpression. Scale bar 100 μm.

**(e**) Embryonic development after microinjection. Different letters in same column indicate significant difference (P < 0.001).

**(f**) Representative immunofluorescence images of p-YAP in control, GAS5 knockdown (siGAS5), and GAS5 overexpressing (GAS5-OE) morula. Images are representative of three independent experiments. Scale bar 50 μm.
**(g)** Quantification of fluorescence intensity. Morula were divided into outer and inner cells. p-YAP intensity in the cytoplasm (Cyt) and nucleoplasm (Nuc) was quantified separately for outer and inner cells. Two-tailed Student’s t-test was used for the statistical analysis. The data are presented as the mean ± SEM.

**(h**) Representative immunofluorescence images of YAP in control, GAS5 knockdown (siGAS5), and GAS5 overexpressing (GAS5-OE) morula. Images are representative of three independent experiments. Scale bar 50 μm.
**(i**) Quantification of fluorescence intensity. YAP intensity in the cytoplasm (Cyt) and nucleoplasm (Nuc) was quantified separately for outer and inner cells of morula. Two-tailed Student’s t-test was used for the statistical analysis. The data are presented as the mean ± SEM.


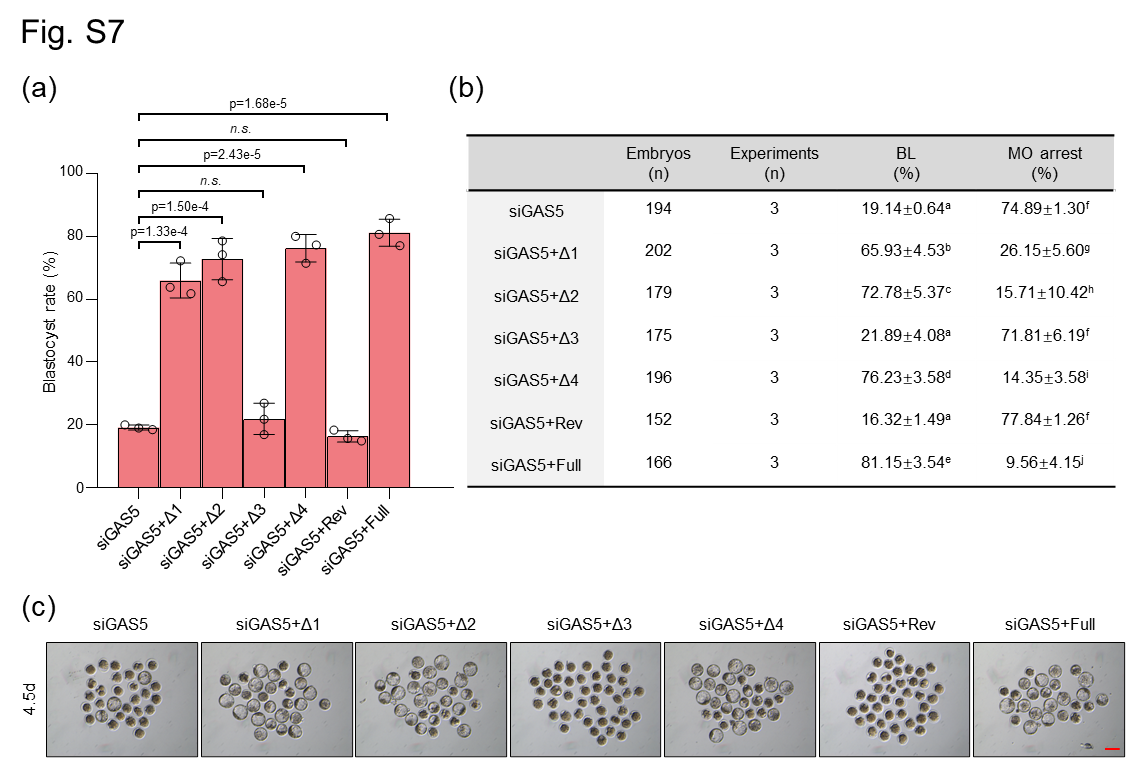


**Fig. S7 Functional rescue of GAS5 depletion requires specific RNA domains**

**(a**) Blastocyst rates upon overexpression of six GAS5 truncation mutants during GAS5 depletion. siGAS5 and siGAS5 + Δ3 significantly reduced blastocyst formation. *n* = 3 biologically independent experiments. Two-tailed Student’s t-test was used for the statistical analysis. The data are presented as the mean ± SEM.

**(b)** Embryonic development after microinjection. Different letters in same column indicate significant difference (P < 0.001). *n* = 3 biologically independent experiments.

**(c**) Morphological phenotypes of embryos overexpressing truncated GAS5 RNAs under GAS5 depletion. Embryos were imaged at 110 h phCG (blastocyst stage). siGAS5 + Δ1/Δ2/Δ4/Mut groups developed to blastocysts, while siGAS5 and siGAS5 + Δ3 arrested at morula stage. Scale bar 100 μm.
